# Cyclodextrin-based rotaxanes for polymer materials: challenge on simultaneous realization of inexpensive production and defined structures

**DOI:** 10.3762/bjoc.20.252

**Published:** 2024-11-19

**Authors:** Yosuke Akae

**Affiliations:** 1 Institute for Chemical Technology and Polymer Chemistry (ITCP), Karlsruhe Institute of Technology (KIT), 76131 Karlsruhe, Germany; 2 Faculty of Textile Science and Technology, Shinshu University, 386-8567 Nagano, Japan; 3 Research Fellow of Japan Society for the Promotion of Science, 102-0083 Tokyo, Japan

**Keywords:** cyclodextrin, defined structure, precise synthesis, rotaxanes, stimuli-responsive material

## Abstract

Owing to their dynamic natures, rotaxane-based polymers are attractive motifs for developing stimuli-responsive materials. However, the accurate control of the rotaxane structure, which can be achieved via multistep synthesis, is key to utilizing the material. Concurrently, implementing a scale-up synthesis procedure to exploit the application potential of rotaxane-based polymers induces structural ambiguities, thereby presenting a significant trade-off between realizing inexpensive production and defined structures. To overcome this rotaxane-synthesis challenge, cyclodextrin (CD) can be employed as a promising alternative owing to its low production cost. Thus, this study presents an overview of the precise synthesis of CD-based rotaxane and its application to polymers to simultaneously ensure inexpensive production and realize defined structures.

## Introduction

**Figure 1:**
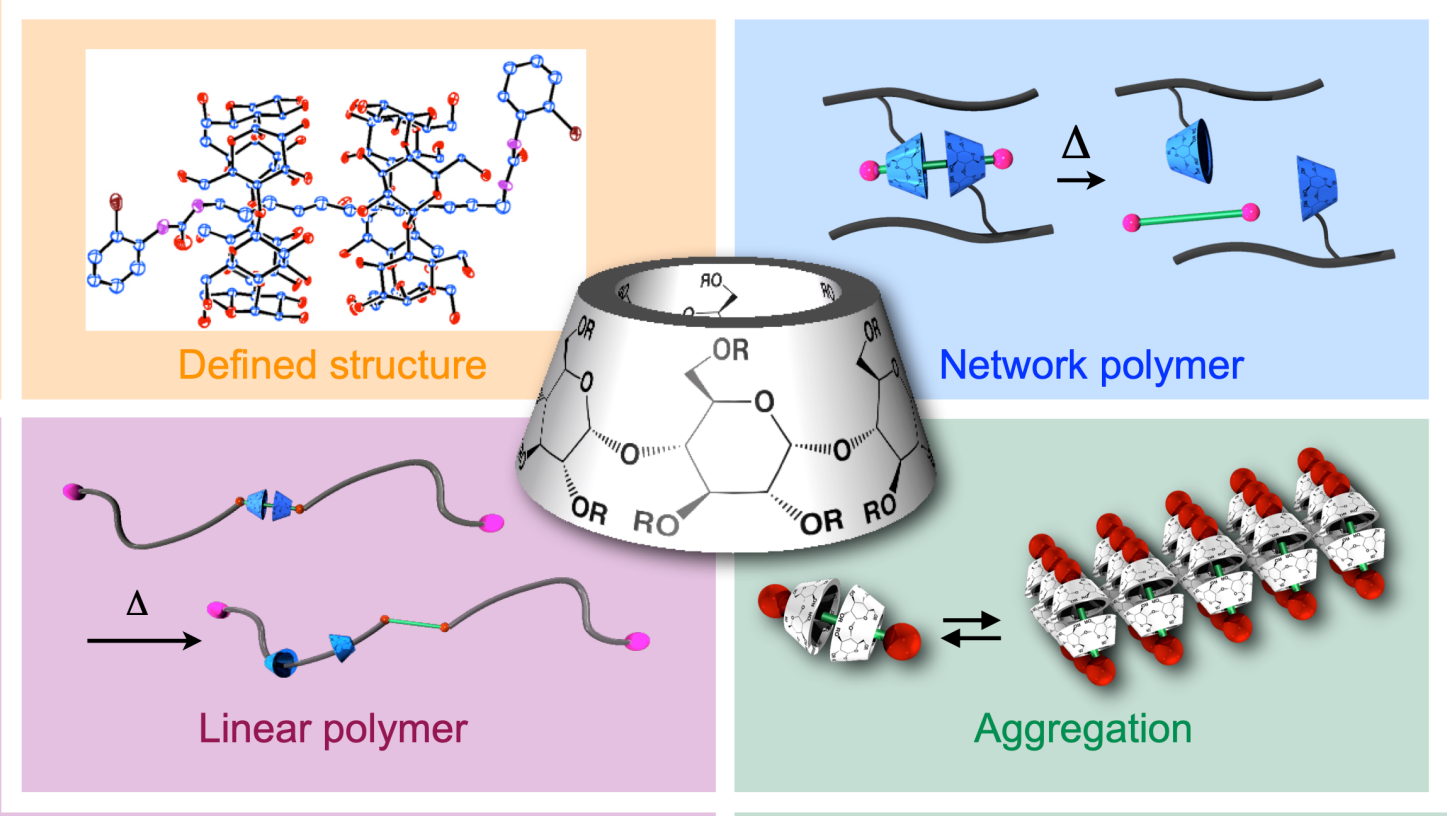
Overview of the CD-based rotaxane as a polymer material covered in this review.

Stimuli-responsive materials can satisfy the existing high demands on such materials owing to their multiple functionalities (e.g., for effective energy storage or as decomposables under mild conditions) that facilitate sustainability [1–10]. In this regard, the rotaxane framework represents an attractive motif for implementing substantial property changes triggered by certain stimuli owing to its higher dynamic nature than those of conventional materials [11–15]. A variety of unique rotaxane-based architectures (e.g., mechano-responsive material, polymers exhibiting pump-like motion, and biomimetic sequential reactors) exhibiting conventionally unaddressed unique properties have been developed based on modern synthetic chemistry [16–30]. To maximize the utility of rotaxane-based architectures, the accurate control of their structures is the key, although multistep synthesis is required to implement an elaborate molecular design. However, realizing the material application requires a scalable synthesis procedure to produce an actual material with masses of ≥100 g, as 100 mg of the product is insufficient. Notably, the scalable synthesis of rotaxane-based polymer materials inevitably introduces structural ambiguity when aiming to reduce the synthetic cost, and this complicates the possibility of maintaining a structural control that is as high as that in the small-molecule system, where complicated synthesis is more accepted. Employing cyclodextrin (CD) as a ring unit represents a reasonable option for reducing the synthetic cost because of its lower production cost compared with those of other typical ring molecules [31–35]. Meanwhile, the studies on CD-based rotaxane are generally divided into two aspects: (1) the construction of a complicated small-molecule framework and the evaluation of its properties, and (2) the development of a polymer material (e.g., polyrotaxane) with a rough structural control. Ideally, the accurate control of the structure of a small-molecule system is directly transferred to the corresponding polymer framework to implement a defined rotaxane structure on the latter for precise property control, although an existing huge gap between the studies on rotaxane-based small molecules and polymer material prevents their further development. Based on the foregoing, the precise control of the structures of small molecules, which applies to polymer systems, is highly desired to implement an elaborate structure–property control in rotaxane-based polymer materials and fabricate polymer materials featuring a defined rotaxane structure. In this review, this issue is discussed in three sections: (1) synthesis and structure of CD-based (poly)rotaxanes, (2) structural control of the (poly)rotaxane framework, and (3) properties and applications of rotaxane-based polymer materials (Figure 1). Although this study mainly explored the CD-based framework, other studies on rotaxanes based on other ring units were occasionally considered for comparison. After exploring the current status of this issue, the challenge and future outlook are also discussed.

## Review

### Synthesis and structure of cyclodextrin-based (poly)rotaxanes

**Figure 2:**
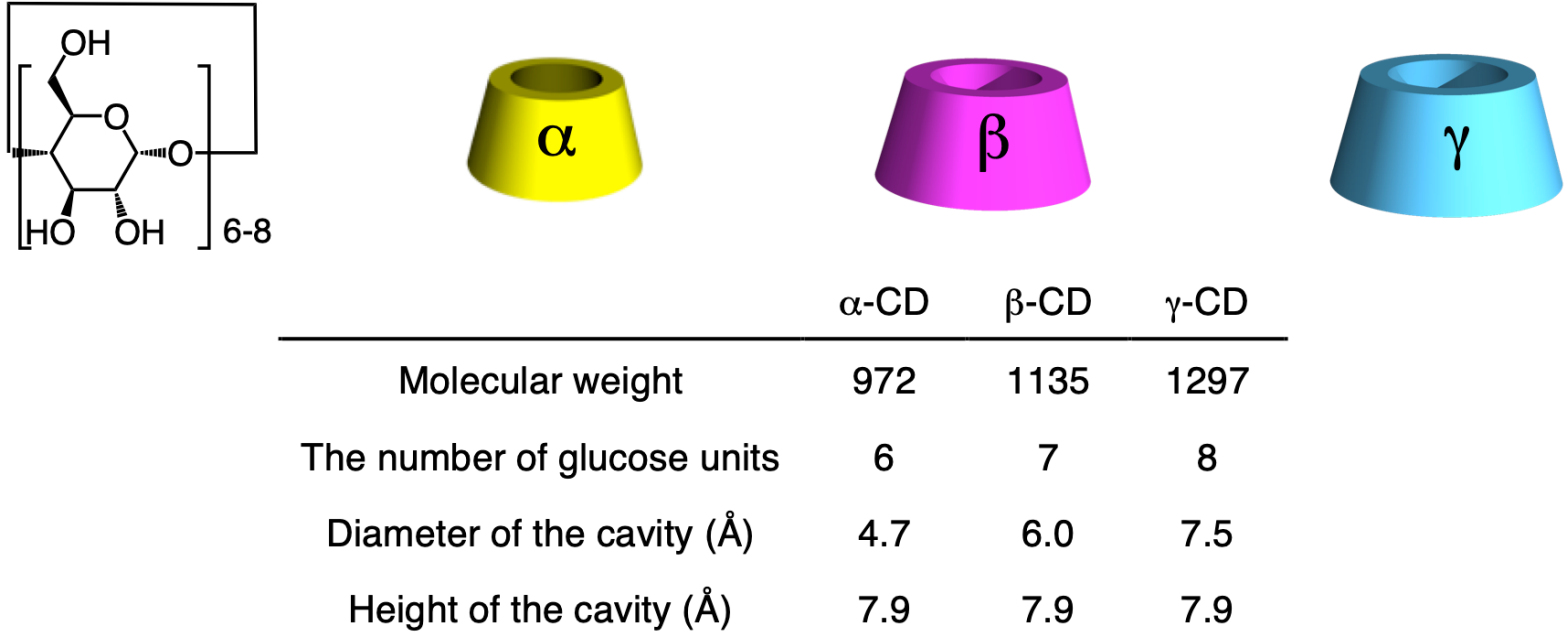
CD structure.

CDs are cyclic sugar molecules in which multiple glucose units that are connected by α-1,4 bonds, e.g., α-CD, β-CD, and γ-CD (glucose-unit numbers 6, 7, and 8, respectively), are used for scientific studies and industrial productions (Figure 2) [31–35]. CDs exhibit the same heights, although the diameters of their cavities differ according to the number of glucose units. As all the hydroxy groups are directly outside the cavity, CDs exhibit hydrophilic and hydrophobic outer and inner structures, respectively, making the CD molecule a good host molecule that interacts with guest molecules via hydrophobic interactions. To maximize the host–guest hydrophobic interaction, suitable guest molecules for CDs generally fit well with their cavity sizes. Put differently, α-CD fits well with a linear aliphatic chain, β-CD does with a substituted benzene ring, and γ-CD can host further bigger guest molecules, such as pyrene or two aliphatic chains, in one cavity. The ability of CD to form an inclusion complex structure is drastically affected by the cavity size or the modification strategy for the hydroxy groups on CD. To evaluate the formation of this inclusion complex, circular dichroism spectroscopy represents a powerful tool when combined with nuclear magnetic resonance (NMR) and X-ray single-crystal analysis. This is because the induced circular dichroism (ICD) can be observed on the guest molecule of the inclusion complex derived from the chiral nature of the CD molecule, and its behavior fits well with the theoretical consideration.

**Figure 3:**
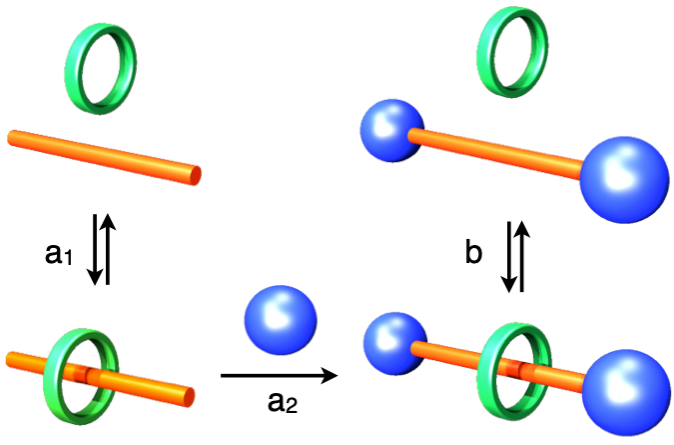
Typical pathway for synthesizing CD-based rotaxanes.

As comprehensively described [32], CD-based rotaxanes are typically synthesized by two methods: (1) the end-capping method (route a_1_, a_2_) in which the formation of a pseudorotaxane structure first proceeds through the formation of CD and an axle molecule, followed by the end-capping of the axle end; (2) the slipping method (route b) involving threading a dumbbell molecule comprising bulky axle-end groups through CD. As the latter is likely to produce a pseudorotaxane framework, the end-capping methods are more popular than the slipping method. However, this end-capping method must fulfill the following seven points to appropriately realize a rotaxane framework, and this causes several complexities (Figure 3): (1) The axle molecule must form a stable inclusion complex with CD, (2) the axle molecule must be sufficiently long to penetrate the CD cavity and be available for the end-capping reactions, (3) the inclusion complex must dissolve appropriately, (4) the solvent must not decompose the inclusion complex (water or other polar organic solvents, such as dimethylformamide (DMF) and dimethyl sulfoxide (DMSO) must be available as CD forms the inclusion complex), (5) the yield of the end-capping reaction must be high, (6) the end-capping reagent and rotaxane must dissolve in the same solvent to facilitate a homogeneous reaction, and (7) the rotaxane must be isolable from the reaction mixture.

**Scheme 1:**
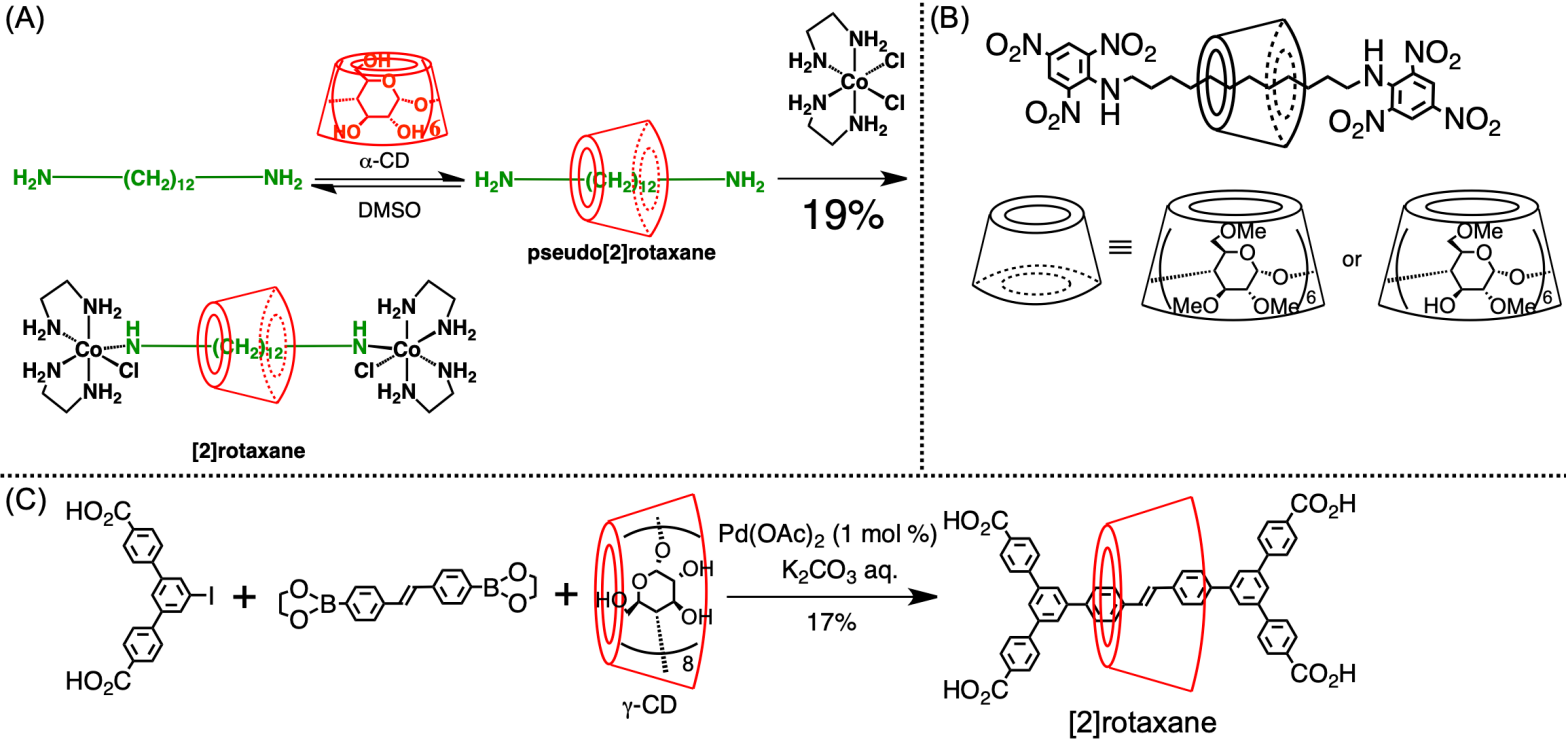
(A) Synthesis of α-CD-based [2]rotaxane via a metal–ligand complex. (B) Chemical structures of methylated α-CD-based [2]rotaxane. (C) Synthesis of γ-CD-based [2]rotaxane in an aqueous solution.

The first example of the synthesis of CD-based rotaxanes was reported by Ogino in 1981 (Scheme 1A) [36]. The hydrophobic interaction between the α-CD cavity and the alkyl chain of α,ω-diaminoalkane yielded the inclusion complex structure, and the ligand exchange between the amino groups on the axle ends and the cobalt complex yielded [2]rotaxane (yield: 19%). Before that, the inclusion complex structure was conventionally formed randomly, resulting in a much lower yield than that obtained by this method and indicating that this 19% yield represented a significant increase compared with that of conventional synthesis. A similar [2]rotaxane molecule bearing a Co complex exhibited ICD derived from the chirality of CD on the absorption band of the cobalt complex [37]. Afterward, numerous CD-based rotaxane syntheses were reported; they generally contained noncovalent-bond moieties on the dumbbell structure in the early days. Rotaxane bearing a dumbbell comprising only covalent bonds was first reported by Harada and co-workers in 1997 (Scheme 1B) [38]. In this system, the end-capping reaction was based on the nucleophilic substitution of the amino groups on the axle ends. Afterward, such nucleophilic substitution/addition reactions became the standard for end-capping reactions, although a transition metal–catalyzed cross-coupling reaction has also been used to synthesize CD-based rotaxane. Typically, water-soluble components are prepared, after which the Suzuki coupling reaction in water is used to synthesize the rotaxane. For example, Anderson and co-workers reported the synthesis of γ-CD–based [2]rotaxane via the Suzuki reaction using a dicarboxylic acid end-capping reagent; they reported a 17% yield (Scheme 1C) [39]. To perform an end-capping reaction in water, multiple hydrophilic substituents, such as carboxylic acid or sulfonic acid groups must be integrated into the system, thus limiting the flexibility of the molecular design of rotaxane. Even after appropriately integrating these hydrophilic groups into the molecular design, the yield of the rotaxanation step is likely to be as low as 10–20% owing to the already described synthesis challenge (Figure 3). In a rough explanation, a 30% yield is already considered high for CD-based rotaxane synthesis, and a 10% yield is sufficient for some specific structural or property analyses, such as electrochemical or optical measurements. To maintain the inclusion complex structure, end-capping is generally conducted under a mild condition, i.e., high temperatures are better avoided, as they limit the reaction options.

**Scheme 2:**
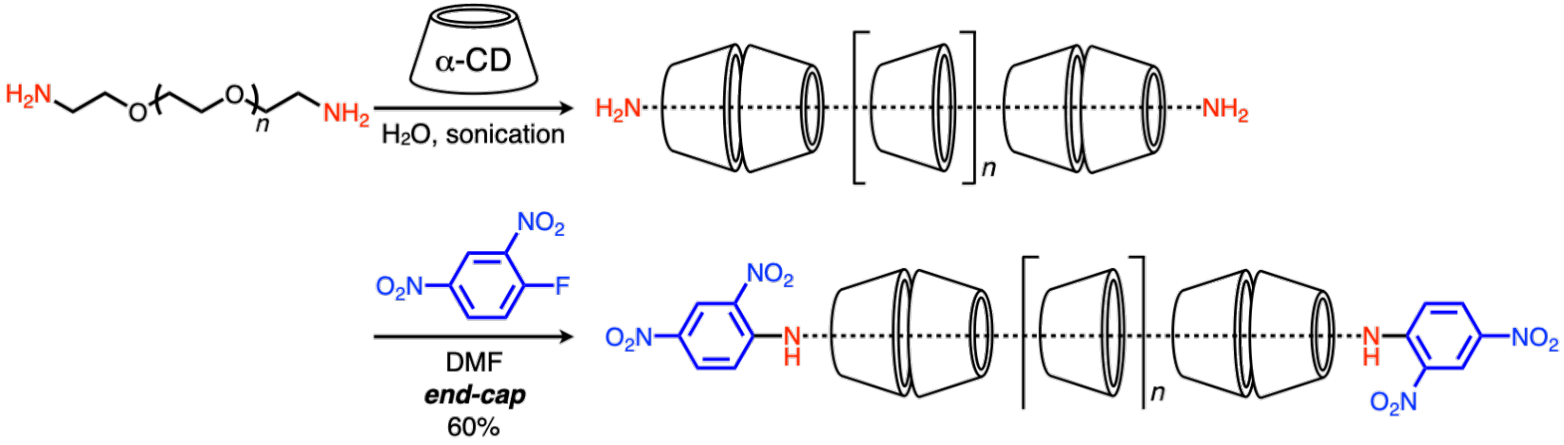
Synthesis of α-CD-based polyrotaxane.

The yield of polyrotaxane synthesis is likely to be higher than that of small-rotaxane-molecule synthesis. As polyrotaxane generally comprises a linear polymer-chain-like polyethylene glycol (PEG) and many rings, the loss of a few rings at the end-capping step is not expected to cause a severe issue during polyrotaxane synthesis, as applicable in small-rotaxane-molecule synthesis. Harada and Kamachi first synthesized pseudopolyrotaxane by simply mixing α-CD and PEG in water [40]. In this reaction, all the components were soluble in water, although the product did not dissolve. This indicated that pseudopolyrotaxane could be collected by the simple filtration of the reaction mixture. This pseudopolyrotaxane synthesis also depended on the cavity size, the same as small-molecule inclusion. For example, PEG forms stable pseudopolyrotaxane structures with α, β, and γ-CDs, although polypropylene glycol (PPG) only forms a stable pseudopolyrotaxane structure with β-CD [41]. In addition to these linear polymer chains, various types of polymers are known to form inclusion complex structures with CDs [42]. Notably, polyrotaxane synthesis is much more complicated than pseudopolyrotaxane synthesis owing to its multiple requirements, as mentioned in the previous section (Figure 3). However, similar to small-rotaxane-molecule synthesis, Harada and co-workers reported the end-capping reaction based on nucleophilic substitution by the amino groups on axle ends (Scheme 2) [43]. Similarly, other highly efficient reactions have been performed as end-capping reactions to produce polyrotaxane [13–15].

In addition to the simple synthetic study, the structural analysis of the obtained (poly)rotaxanes was performed to evaluate the fundamental relationship between the axle length and the number and directions of the CD wheels. The directions of the CDs on the PEG-based polyrotaxane framework were analyzed by scanning tunneling microscopy (STM) [44], clarifying that 80% of the CDs formed the head-to-head and tail-to-tail repeating patterns in which the wheels were located next to each other on the same cavity side, whereas 20% of the CDs exhibited the head-to-tail structure. This direction was induced by the different strengths of the hydrogen bonding by the primary- or secondary-alcohol groups, as confirmed by theoretical analyses. This structure was consistent with a previously reported X-ray crystal structure analysis of the complex structure comprising α-CD, polyion, and Li^+^ or Cd^2+^ [45]. Regarding the small rotaxane molecule, it was confirmed that a pseudorotaxane comprising an oligo alkyl chain and α-CD contains one CD unit for the six methylene chains. However, further details about the combination of the simple alkyl chain and CD were not clarified probably because of the synthetic complications associated with the end-capping reaction. As a pseudorotaxane structure could be decomposed during measurements (e.g., a solution-based NMR measurement), a more stable rotaxane structure is preferred for the analysis.

**Scheme 3:**
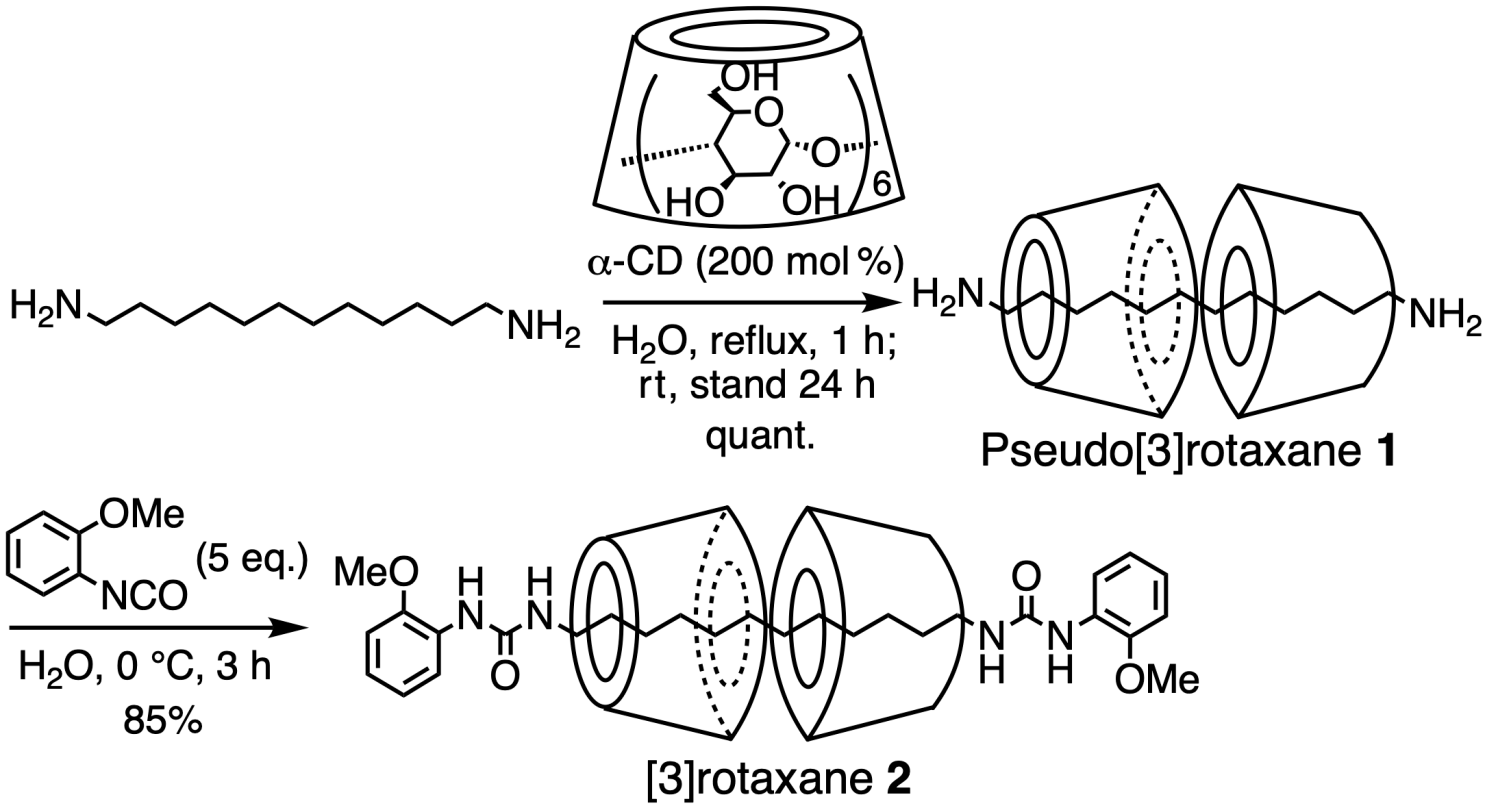
Facile [3]rotaxane synthesis by the urea end-capping method.

Therefore, we developed a facile [3]rotaxane synthesis method based on a urea end-capping method to produce simple structured rotaxane species (Scheme 3) [46–48]. In this method, dodecanediamine and α-CD are mixed in water to form a pseudo[3]rotaxane (**1**), and the subsequent one-pot end-capping reaction driven by the amino group on the axle ends and substituted phenyl isocyanate produced [3]rotaxane in a high yield. In this reaction, the size of the end-capping agent is the key, as an extremely large reagent does not react well with the amino axle-end groups, whereas an extremely small reagent cannot function as an end-cap. This reaction exhibits the following multiple advantages compared with other synthetic methods: (1) the reaction can proceed on a 100 g scale in a 1 L flask and a 500 mL solution of water, sufficiently indicating the possibility of large-scale material applications; (2) the product contains only head-to-head structured [3]rotaxane, as confirmed by the X-ray crystal structure analysis, and is purified by simple precipitation without requiring chromatography; (3) various end-capping agents are available, and well-optimized conditions can ensure a high yield (e.g., 2-methoxyphenyl isocyanate obtains an 85% yield after 3 h of reaction [49]). Owing to such a high-yield reaction via a simple process compared with other synthesis methods, this method can be developed as a platform for constructing rotaxane structures for further supramolecular-architecture designs, which will be explored in more detail.

**Figure 4:**
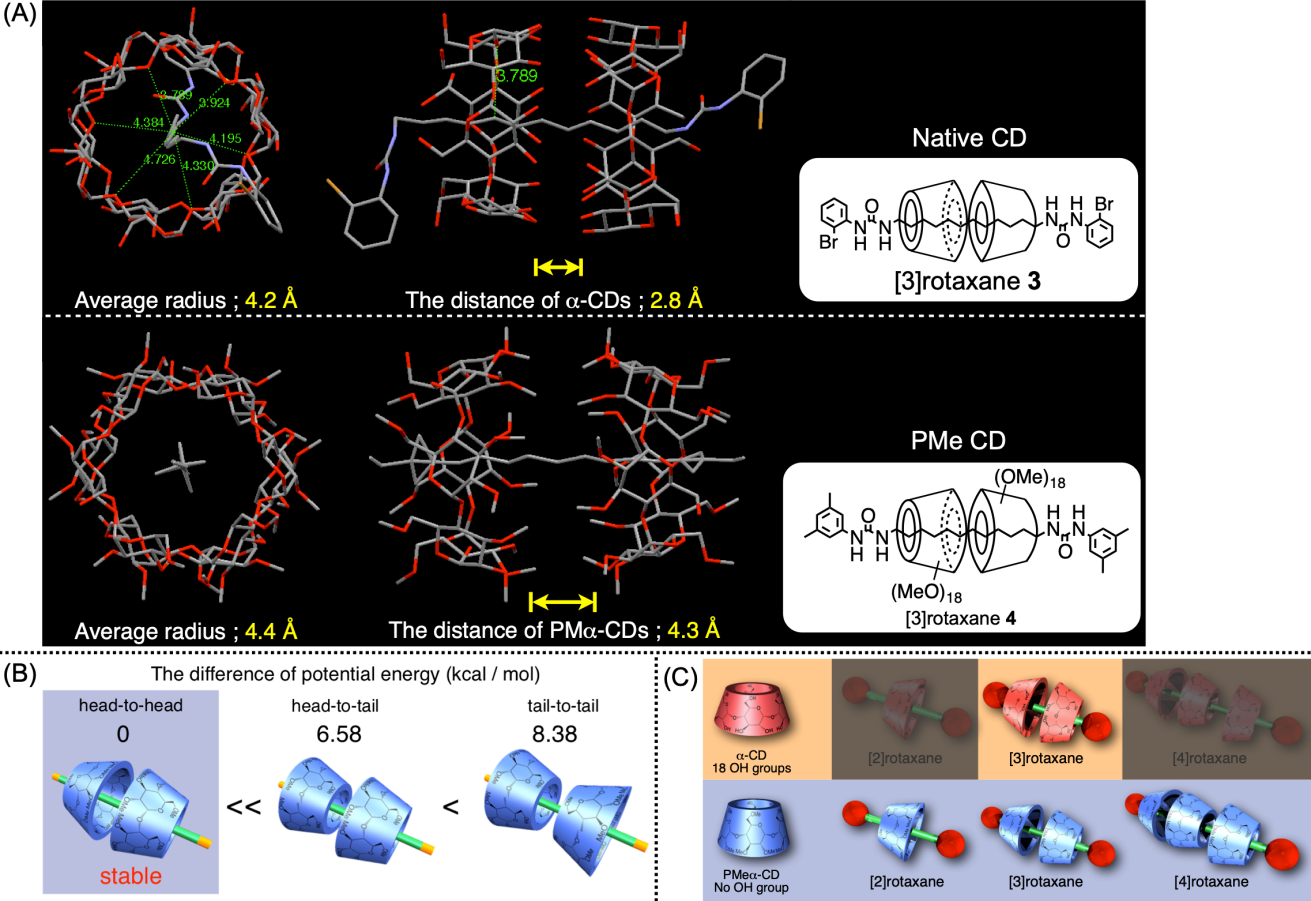
(A) Single-crystal structure of α-CD-based [3]rotaxane **3** and PMα-CD-based [3]rotaxane **4**. (B) Schematic of the head-to-head–, head-to-tail–, and tail-to-tail–structured PMα-CD–based pseudo[3]rotaxane and their potential energy difference calculated by molecular dynamics. (C) Comparison of the α-CD-based and PMα-CD-based rotaxanes synthesized from α,ω-diaminoalkane as the axle unit [50–51].

When a perfectly methylated α-CD (PMα-CD) is used for rotaxane synthesis rather than native α-CD, the rotaxane molecules exhibiting an odd number of wheel units would be obtained, whereas native α-CD only produces an even number of wheel units (Figure 4) [50–51]. These phenomena have been comprehensively studied using multiple α,ω-diaminoalkane bearing 8, 10, 12, 18, or 24 methylene units as the axle component. The results revealed that the weakened hydrogen bonding and hydrophobic interaction by PMα-CD was the key to the formation of rotaxane molecules with an odd number of wheel components as well as their much lower yields than the native-CD-based rotaxanes. Moreover, owing to the interaction between the narrower rim side of PMα-CD and the amino groups on the axle ends, the [3]rotaxane- or [4]rotaxane-species-contained PMα-CDs directed their narrower rim sides to the axle ends, as confirmed by theoretical calculations, thus indicating that such relatively weak interactions could fix the rotaxane structure. Such a fundamental systematic study was first possible because of the efficient urea end-capping synthetic method that supported its utility.

Notably, other types of rotaxane frameworks have been developed using the pseudo[3]rotaxane-formation or urea end-capping method [52–55]. For example, a CD-based [3]rotaxane framework was found to be an effective motif for organ imaging by combining it with a metal complex owing to the densely placed hydroxy groups [52].

As described above, the synthesis methods for CD-based (poly)rotaxane species have been considerably studied. However, in a small-molecule system, high-yield synthesis is still not so conventional except with the urea end-capping method. In the next section, the structural regulation system is explored and discussed in terms of its applicability to polymer systems.

### Structural control of the (poly)rotaxane framework

The rotaxane framework is often deployed as a molecular device in which the positional relationship between the axle and wheel is controlled by external stimuli [14,32]. Acid–base, photoisomerization, redox, ion recognition, solvent polarity, or heating treatment is generally used for such stimuli. When the rotaxane motif contains cyclophane or a crown ether, stimuli induced by a transition metal or an electrostatic interaction have been widely used. These interactions are easily controlled, and they induce relatively strong coordination interactions among the components, making them suitable media for regulating the rotaxane structure. Concurrently, for CD-based rotaxane systems, employing a transition metal or inducing electrostatic interactions encounters more limitations owing to the synthetic shortcoming of integrating high-polar moieties in their dumbbell framework that is derived from the hydrophobic-interaction-based inclusion formation; thus they are only low number of reports on their structural control. The photoisomerization-based structural control system is often applied to CD-based rotaxane. Typically, the photo-switch behavior is used, and it is based on the relationship between the photoisomerization of azobenzene or stilbene and CD to form the inclusion complex structure.

**Figure 5:**
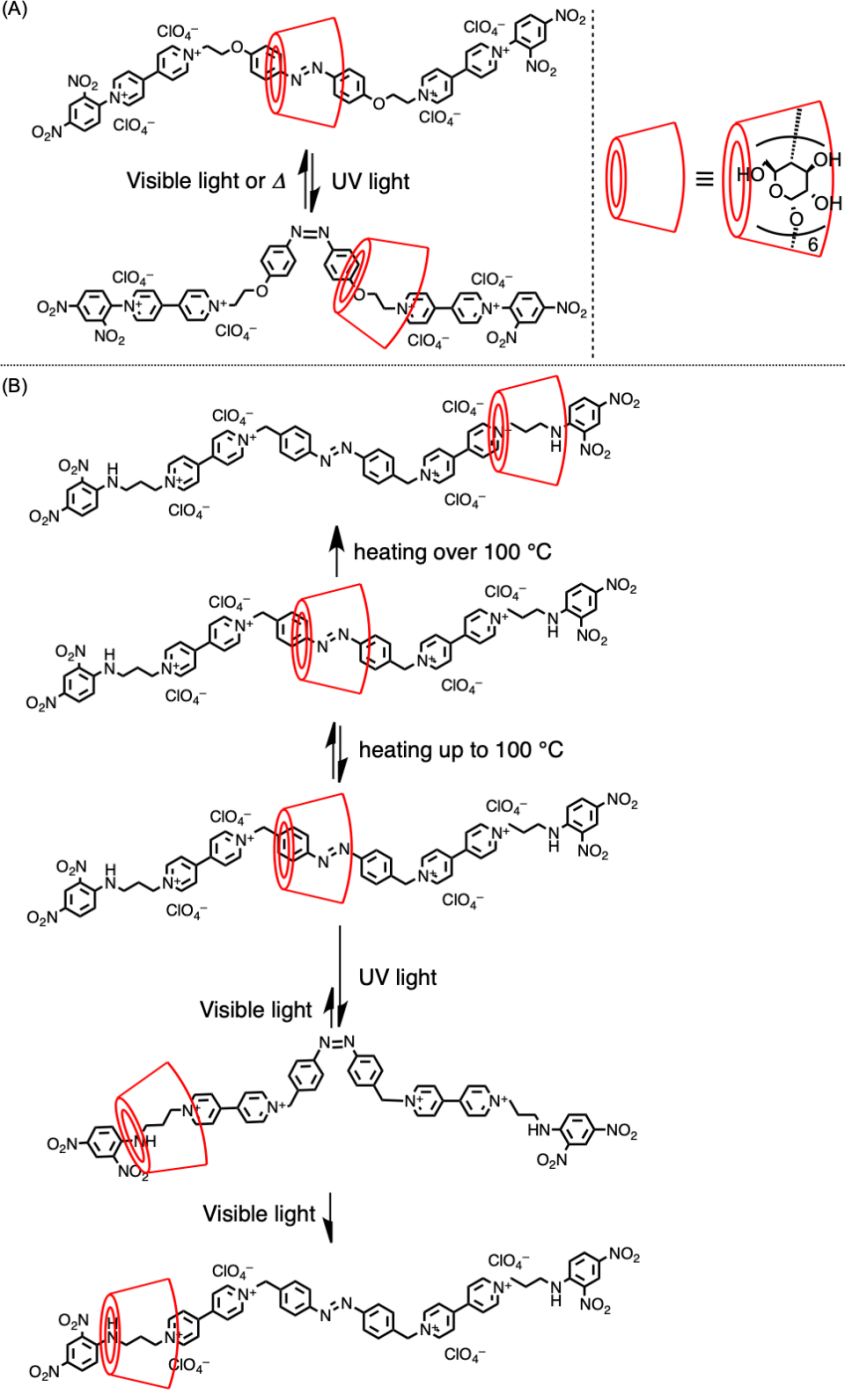
Structural control of CD-based [2]rotaxane via (A) light irradiation and (B) light irradiation and heating.

In 1997, [2]rotaxane exhibiting an azobenzene moiety in its axle component was synthesized, and its structural control was achieved via the photoisomerization of the azobenzene (Figure 5A) [56]. In the structure, α-CD was located on the *trans*-azobenzene moiety before ultraviolet (UV) irradiation, after which it moved to the methylene moiety based on the *cis*-isomerization of the azobenzene by UV irradiation. After the azobenzene moiety was moved back to the *trans*-isomer via visible-light irradiation or heating, CD was moved back to the azobenzene moiety, and the entire process was reversible. This system was further developed in 2005 by introducing bipyridinium moieties to implement the selective transfer of the wheel via heating or light irradiation (Figure 5B) [57].

**Figure 6:**
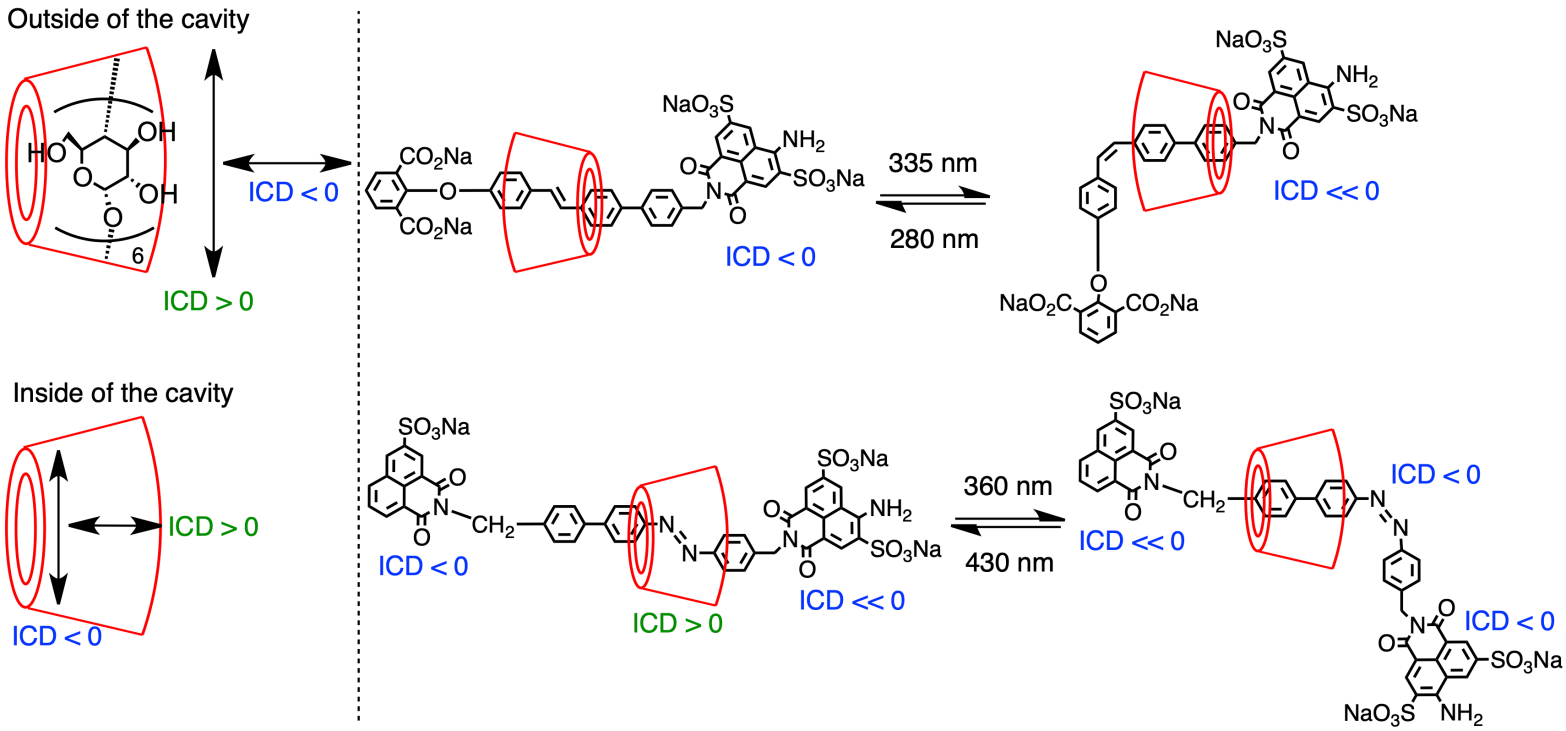
Relationship among the plus–minus signs of ICD, the position of the guest molecule, and the axis of the dipole moment of the guest molecule (left). Chemical structures of α-CD-based [2]rotaxanes and their plus–minus ICDs on each dye moiety (right).

During such structural regulation of the rotaxane framework, NMR was deployed as a very powerful evaluation method, especially the nuclear Overhauser effect or rotating-frame Overhauser effect, which is used to analyze the spatial adjacency of the components. It provides critical data for detecting the position of the wheel on the axle component. Meanwhile, the ICD evaluation based on the chirality of CD is a powerful tool for analyzing the inclusion behavior, which is also used for structural analysis. In 2006, Tian and co-workers reported the structural control of the rotaxane framework by subjecting a α-CD-based rotaxane comprising a stilbene axle that was synthesized by the Suzuki coupling reaction to light irradiation (Figure 6, right) [58]. In this structure, α-CD was first located on the *trans*-stilbene moiety, after which it moved to the benzene ring moiety via the *cis*-isomerization of the stilbene framework by 335 nm light irradiation before returning to the stilbene moiety via *trans*-isomerization induced by the 280 nm light irradiation; this system is also reversible. The change in the local position of CD is significantly reflected in the ICD behavior of this system. Kodaka and co-workers reported that in the case of the dye molecule existing inside the CD cavity, the plus Cotton effect was observed when the dipole was directed along the axle of the cavity, whereas it became minus when the dipole was directed perpendicular to this axle (Figure 6, left) [59]. When the dye molecule was located outside the cavity, these plus and minus signs were transformed into their opposite signs. The structural transformation implemented in Tian’s study displayed consistent ICD with this rule, indicating the utility of this method in CD-based rotaxane systems. When azobenzene was used instead of the stilbene framework, the same consistency was observed [59]. Meanwhile, a partial inclusion of the guest molecule by CD induced irregular ICD patterns, and this phenomenon has not been clarified; thus, further detailed studies are desired [60]. As introduced here, the photoisomerization of azobenzene or stilbene has been generally used to control the position of CD in the rotaxane structure [14–15].

**Figure 7:**
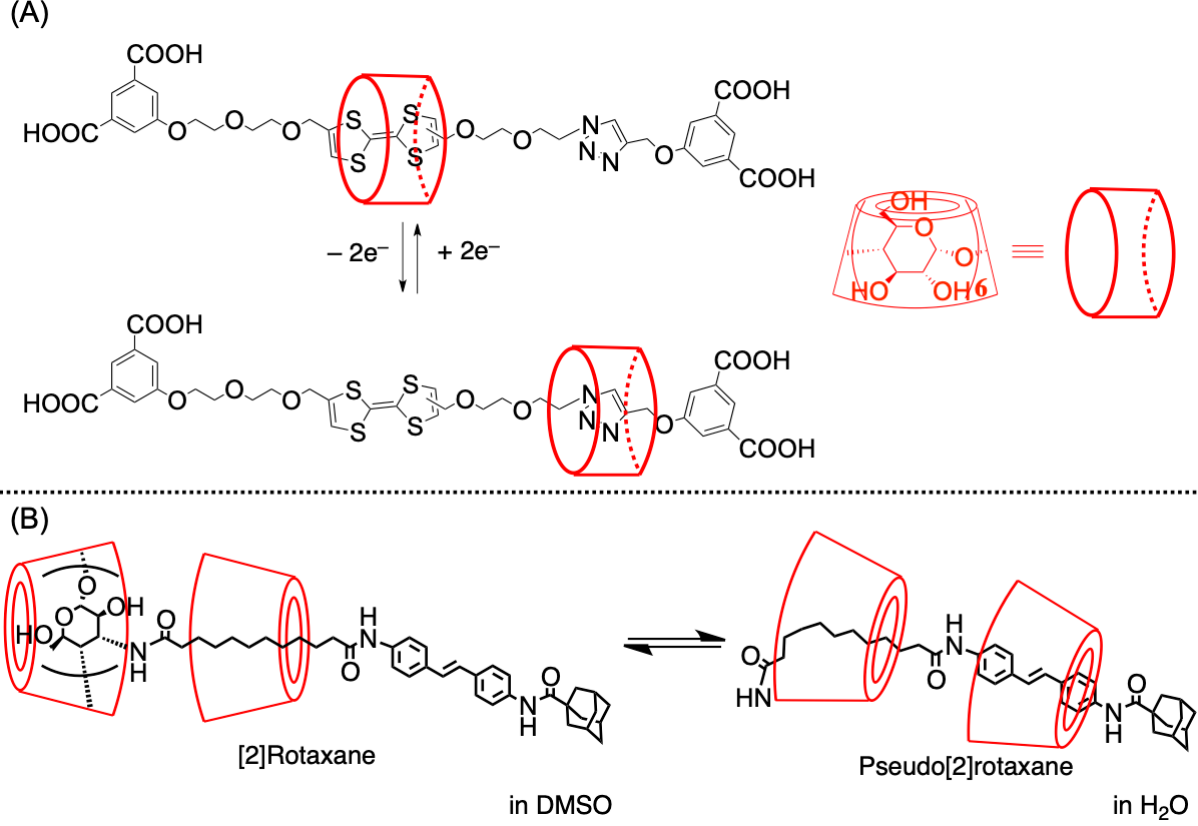
Structural control of CD-based rotaxane via (A) redox reaction and (B) in a solvent.

In addition to the photoisomerization–based system, redox-based structural control was reported by Stoddart and co-workers in 2008 using α-CD-based [2]rotaxane bearing a dumbbell molecule comprising tetrathiafulvalene (TTF) and a triazole ring (Figure 7A) [61]. CD was located around the TTF moiety under neutral conditions; however, it moved to the triazole ring, following TTF oxidization. Meanwhile, the structural control by exploring the solvent polarity was reported by Harada and co-workers using the α-CD-based [2]rotaxane exhibiting α-CD as the end-capping moiety (Figure 7B) [62]. The wheel was located on the methylene moiety in DMSO, although it moved to the stilbene moiety in water via inclusion formation between CD on the axle-end moiety and the methylene chain. When [3]rotaxane exhibiting another wheel on the stilbene moiety was used for this system, no structural change was induced by the change in the solvent. The self-inclusion of [2]rotaxane in water was induced by the hydrophobic interaction.

As shown above, various structural regulation systems were developed for CD-based rotaxane. However, most of their rotaxane structures required a complicated multistep synthesis as well as the introduction of polar substituents, such as carboxylic acid or sulfonic acid groups, to generate a water-soluble system, and these prevent the direct application of those frameworks to polymer systems. As the defined structural control of rotaxane-based polymer systems could induce drastic property changes in the material, its implementation presents an interesting scientific issue. Meanwhile, the control of the coverage ratio of the CD units on the polyrotaxane, i.e., the number of CDs on the axle-polymer component, is not so easy because of the strong hydrogen bonding between the CD units. Beckham and Zhao reported that an increase in the molecular weight of the axle polymer caused a decrease in the coverage ratio, although the coverage ratio in the same molecular weight polymer could not be controlled at first [63]. In such structural-regulation development in the polymer system, the approach has been different from that for small-molecule systems probably because of the synthetic complications involved in implementing the structural control for small molecules to the polymer system.

**Scheme 4:**
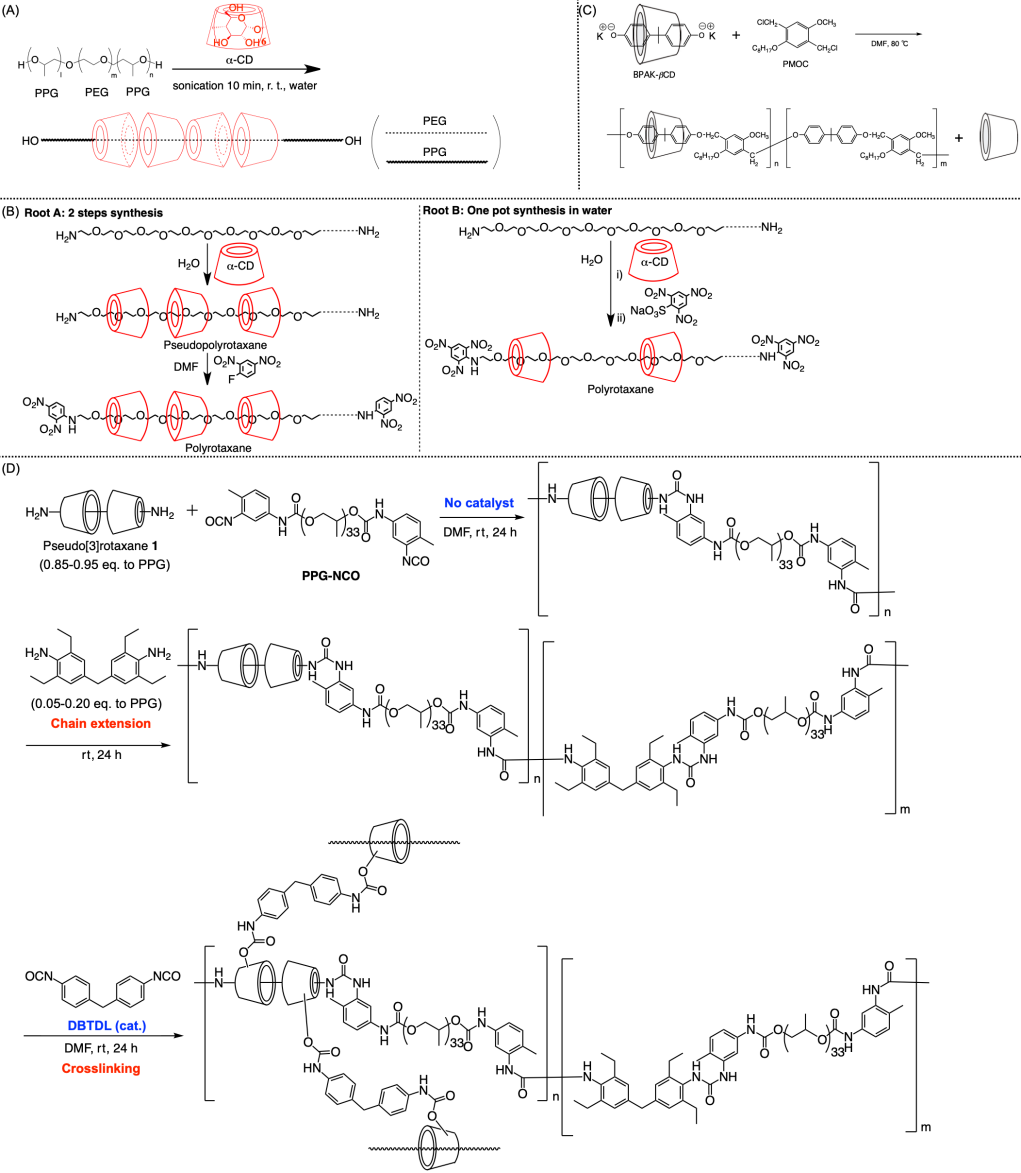
(A) Synthesis of pseudopolyrotaxane bearing an ABA triblock copolymer as an axle. (B) Two synthetic roots for controlling the coverage ratio of polyrotaxane. (C) Polyrotaxane synthesis via the polycondensation reaction between pseudo[2]rotaxane and a bulky comonomer. (D) Low-covered polyrotaxane synthesis via polyaddition between **1** and a (macro)comonomer.

In 2001, Li and co-workers reported that pseudopolyrotaxane, which was obtained by mixing the PPG–PEG–PPG ABA-triblock copolymer and α-CD in water, contained CDs only on the PEG moiety (Scheme 4A) [64]. In 2005, Hadziioannou and co-workers reported the synthesis of pseudopolyrotaxane containing high-molecular-weight PEG (*M*_n_ = 20,000), revealing that the average number of penetrated CDs could be controlled from 3 to 125 (theoretical maximum was 227) by adjusting the concentration ratio of α-CD to PEG, the reaction temperature, and the end-capping method (Scheme 4B) [65]. Concurrently, in 2007, Jouini and co-workers reported the coverage-ratio regulation achieved by controlling the feed ratio of β-CD-based pseudo[2]rotaxane and the spacer comonomer through copolymerization (Scheme 4C) [66]. Similarly, we reported the synthesis of poly[3]rotaxane via the polyaddition reaction of pseudo[3]rotaxane diamine and di-isocyanate comonomers (Scheme 4D) [67]. In this polyaddition reaction, various combinations of comonomers, e.g., di-isocyanate macro comonomers, bulky comonomers, or bulky diamine comonomers, enabled the fine-tuning of the resulting polymer properties. In addition to one-shot polymerization, chain extension and the cross-linking of the hydroxy groups on CDs could extend the molecular/material design. As the di-isocyanate macromonomer was used for long axle components, the coverage ratio of CDs in this system was 3–5%, which was significantly lower than those of other systems in 2020. The mechanical properties of this poly[3]rotaxane exhibiting a *M*_w_ of ≈100,000 [g/mol] (Young’s modulus, 7.1 MPa; fracture strain, 560%; fracture stress, 4.6 MPa) reached a practical level as an industrial material. Moreover, thanks to the facile synthesis of the pseudo[3]rotaxane comonomer, as aforementioned, the 100 g-scale synthesis was not challenging for this material, deviating significantly from the existing methods for synthesizing poly[3]rotaxanes.

As shown here, the control of the coverage ratio of the CD-based polyrotaxane has been challenging, and its regulation method using the desired molecular weight of the axle polymer has been desired. To resolve this issue, utilizing a mixed solvent at the inclusion-formation step was considered an option. For the pseudopolyrotaxane comprising poly(caprolactone) and α-CD in a DMF/water mixed solvent, the increase in the DMF caused a decrease in the coverage ratio and product yield, and these were consistent with the general trend [14,68]. As reported here, a typical coverage-ratio control of the CD-based rotaxane framework has been implemented only at the level-containing distribution, and it has been challenging to realize a defined structure, such as the exact one or two CD units on the polymer chain. This is understandable considering the challenge of the direct transfer of well-developed elaborate structural-regulation methods on small rotaxane molecules to polymer systems. However, the precise structural regulation of rotaxane-based polymers is key to maximizing the utility of the rotaxane framework. To increase the structural control, the development of another controlling method in small rotaxane molecules, which applies to the polymer system, is desired.

**Scheme 5:**
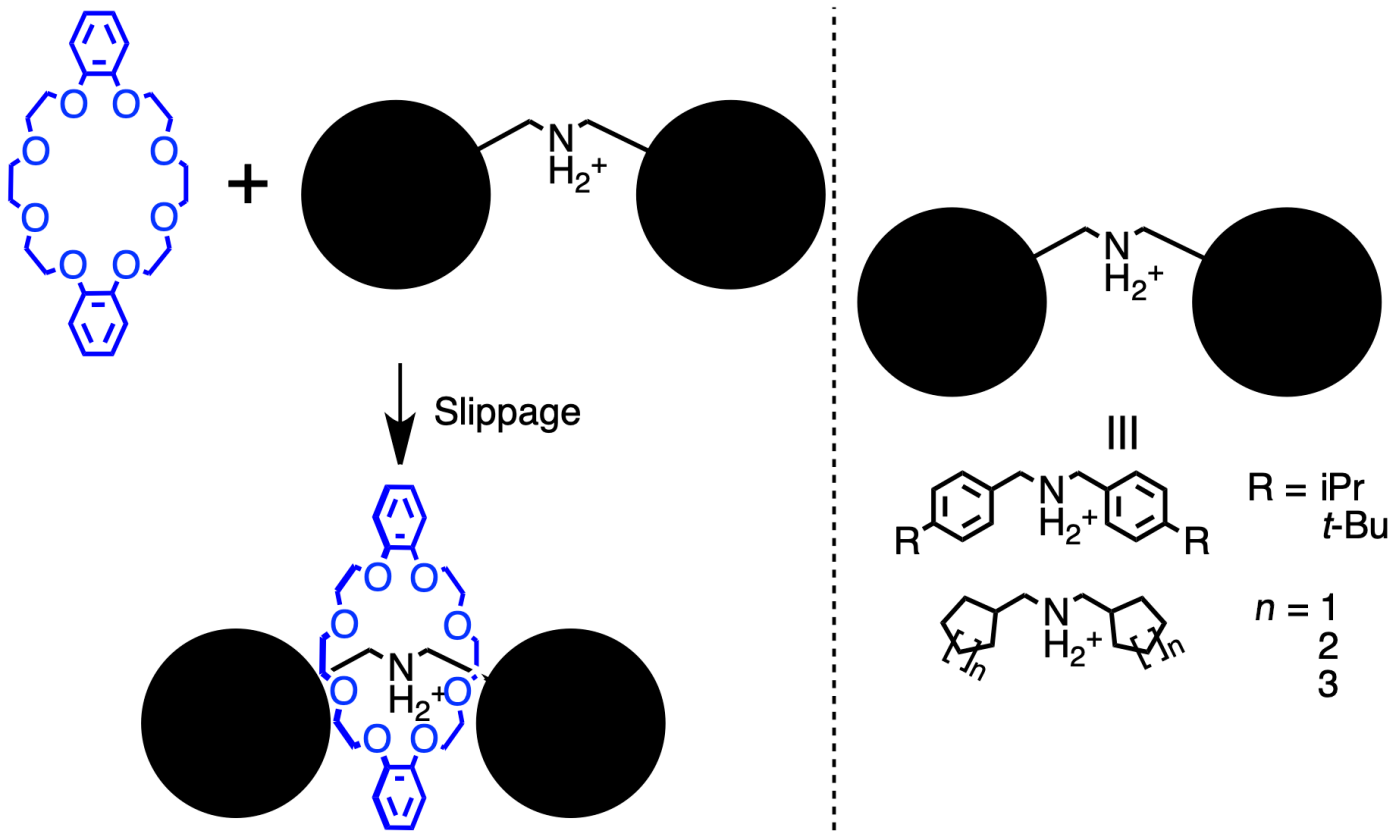
Slippage of size-complementary rotaxanes.

Size-complementary rotaxanes have been considered a candidate for such a structural-regulation system. A size-complementary rotaxane contains axle-end groups that are as large as the wheel cavity. Thus, it is sufficiently stable for isolation under ambient conditions, although a certain stimulus, e.g., heat or the addition of a base, can induce the deslipping reaction to degrade itself as each component. The main issue with utilizing size-complementary rotaxanes is the precise size design on the axle end and wheel cavity, and this indicates that it could be applied to the CD-based polymer system, as it does not require any specific polar or ionic motif in the axle framework. As mentioned in the previous section, the most popular structural-regulation method for the CD-based small-rotaxane framework is the precise control via the photoisomerization of the guest molecule, whereas that of the polymer system would be the rough control based on the change in the solvent composition. Thus, size-complementary rotaxanes offer another option for controlling the structure of CD-based rotaxanes. Although only a few size-complementary rotaxanes have been reported, even for those comprising other ring molecules, some examples are introduced below. In 1998, Stoddart and co-workers reported pseudo[2]rotaxanes comprising dibenzo-24-crown-8 and multiple dumbbell species bearing secondary ammonium salt moieties on their center (Scheme 5) [69]. These species were completely stable when PF_6_^−^ was deployed as a counter-anion. Other than that, they degraded under high-temperature conditions. The stability of the pseudo[2]rotaxane species depended on the bulkiness of the axle-end moieties; thus, some of them exhibited similar stability as rotaxane, whereas others did not, and this introduced ambiguity in defining the pseudorotaxane structure. This could be regarded as the first report on size-complementary rotaxanes. Thereafter, Takata and co-workers integrated this size-complementarity into the asymmetric framework to implement one-direction deslipping movement on the size-complementary [2]rotaxane [70].

**Figure 8:**
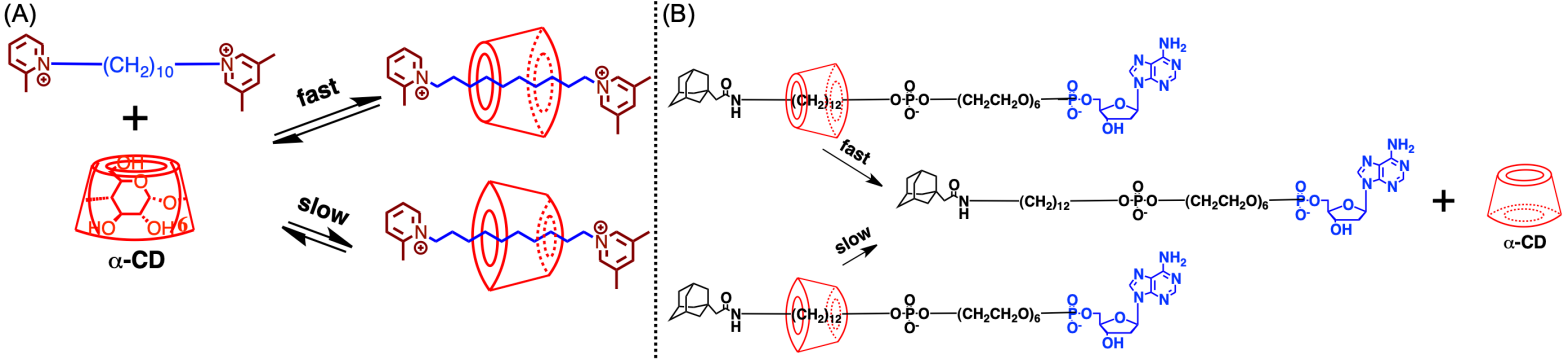
(A) Reversible formation of the CD-based [2]rotaxane. (B) Deslipping reaction of the CD-based size-complementary [2]rotaxane.

Until 2010, only a few examples of CD-based size-complementary rotaxanes have been reported. Harada and co-workers reported the pseudorotaxane equilibrium observed by mixing α-CD and the axle molecules bearing methylpyridinium moieties on their ends, where the bigger rim of CD (the head side) was likely to include the dumbbell (Figure 8A) [71–72]. Concurrently, Komiyama and co-workers performed the kinetic analysis of the deslipping reaction of [2]rotaxanes exhibiting a deoxynucleotide on the axle-end structure (Figure 8B) [73]. They clarified that the deslipping proceeded faster from the bigger rim side of the CD cavity, as expected. However, the direct utilization of those frameworks in the polymer system is challenging owing to the introduction of unconventional functional groups, such as pyridinium or deoxynucleotide. For a flexible integration with the polymer system, a size-complementary rotaxane comprising a simple structure is desired.

**Figure 9:**
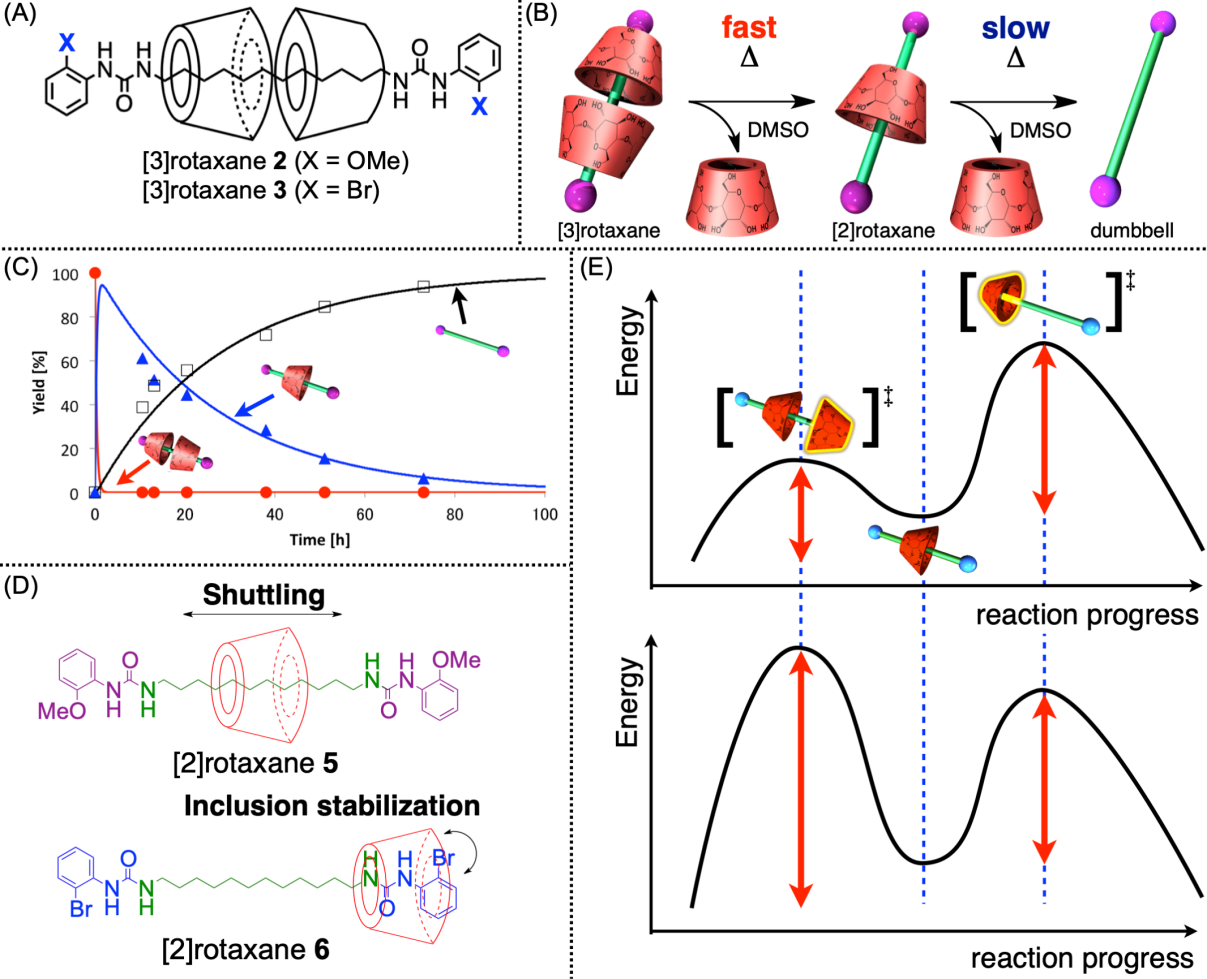
(A) Chemical structures of [3]rotaxanes **2** and **3**. (B) Schematic of the deslipping reaction of [3]rotaxanes **2** and **3** induced by heating in DMSO. (C) Time–yield curves of the degradation of [3]rotaxane **3** in DMSO (8.5 mmol L^−1^ at 353 K). (D) Stabilization effect on [2]rotaxanes **5** and **6**. (E) Energy profile of the stepwise degradation of [3]rotaxane (top) and the direct complete degradation (bottom).

Based on the foregoing, we developed size-complementary rotaxanes via the urea end-capping synthesis method (Figure 9) [46–47]. First, [3]rotaxane 3 synthesized using 2-bromophenyl isocyanate (yield: 73%) was confirmed to be a size-complementary rotaxane whose desilippage was observed in a DMSO solvent by heating at approximately 80 °C (Figure 9A,B). As the rotaxanation step proceeded in water, the product included only [3]rotaxane species owing to the hydrogen bonding among the CD units and axle-end moieties. However, through this deslipping reaction, the corresponding [2]rotaxane **6** was obtained in a 54% yield. The thermodynamic analysis of this deslipping reaction confirmed that the deslipping of [3]rotaxane **3** was approximately 10 times faster than that of [2]rotaxane **6**, offering a local maximum of the yield of [2]rotaxane species in the developing process (approximately 80% by the theoretical calculation) and enabling the isolation of [2]rotaxane during the deslipping-based conversion process [46]. Moreover, considering the similar activation entropy values, the faster deslipping of [3]rotaxane was mainly caused by its lower activation enthalpy than that of [2]rotaxane owing to the stabilization effect derived from the inclusion structure formation from the wider rim side of CD to the axle-end moiety on the latter (Figure 9D). As only the narrower rim side of CDs is directed to the axle-end side on [3]rotaxane **3**, this inclusion formation does not proceed on the [3]rotaxane **3**, thereby inducing the different deslipping speeds between [3]rotaxane and [2]rotaxane. Additionally, when a methoxy group was integrated instead of the bromo substituent, the deslipping of [3]rotaxane **2** became faster and that of [2]rotaxane **5** became slower, thereby inducing a huge rate difference to realize a 94% yield of the theoretical maximum of [2]rotaxane **5** (Figure 9C) [47]. Regarding the methoxy substituent, no inclusion formation was observed in the [2]rotaxane framework, whereas a shuttling motion of the wheel was observed on the axle moiety, indicating that a different stabilization effect was exerted based on the axle-end structures (Figure 9D). Such a stabilization effect could be affected by the bulkiness and polarity of the axle-end moieties. Meanwhile, the deslipping reaction of some [3]rotaxanes directly yielded the dumbbell and two wheels without any [2]rotaxane intermediate, indicating that the deslipping on [2]rotaxane proceeded faster than on [3]rotaxane. In this case, the energy diagram of the deslipping reaction differs from those of the ones bearing a [2]rotaxane intermediate (Figure 9E). As revealed in this study, the CD-based size-complementary rotaxane exhibiting a simple framework (no ionic substituents nor deoxynucleotide) was obtained, and its dynamic nature was comprehensively clarified by thermodynamic analysis. This motivated us to integrate this motif into the polymer system. Further details of the thermodynamic analyses with the actual kinetic parameters are discussed in the original articles [46–47].

**Figure 10:**
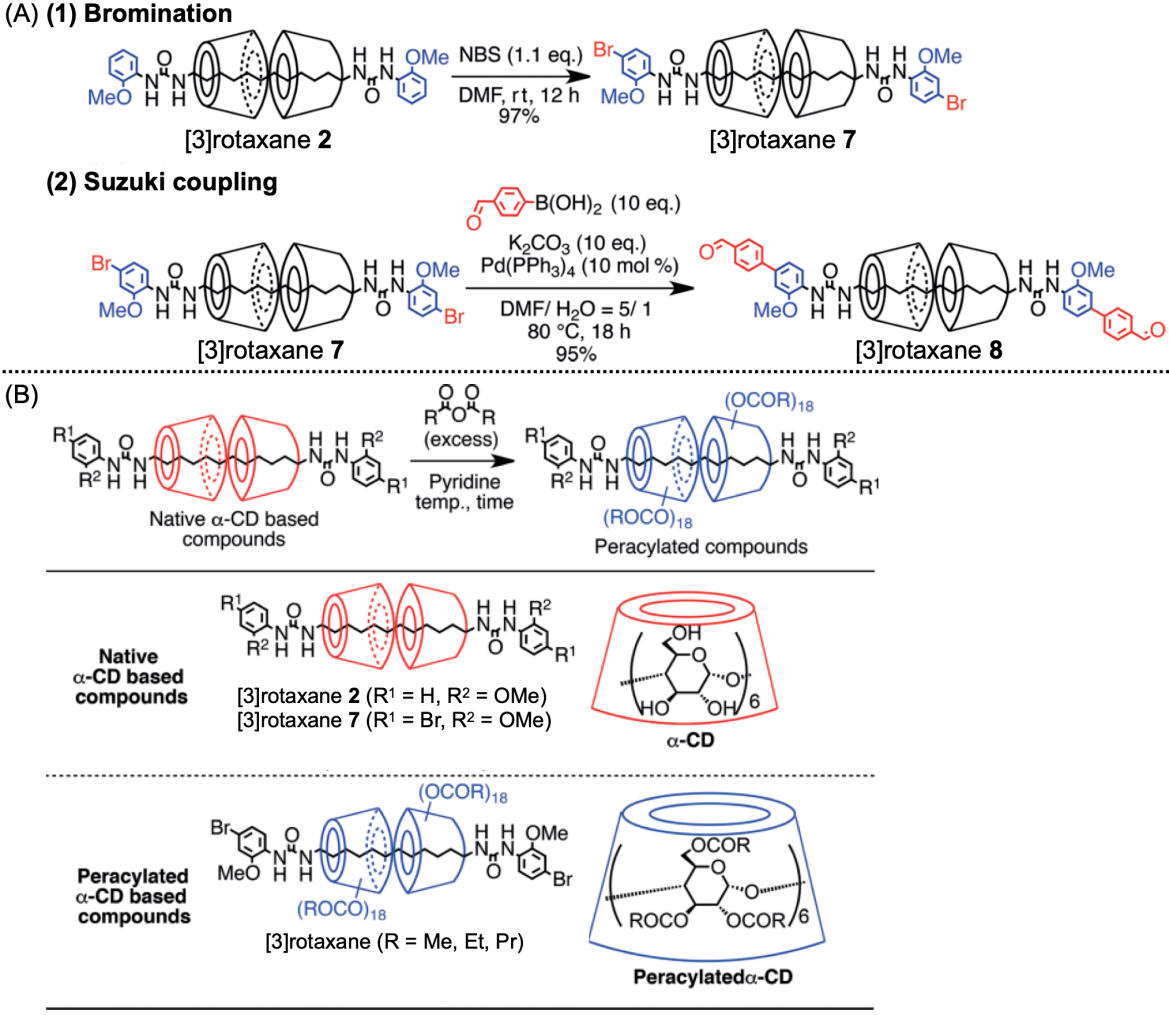
(A) Modification of the axle ends of [3]rotaxane by (1) bromination and (2) the Suzuki coupling reaction. (B) Acylation of the hydroxy groups on [3]rotaxanes and α-CD.

To integrate this motif into the polymer system, further modification and structural analyses were performed [48,74]. The axle end was easily modified by bromination of the benzene ring and successive transition metal–catalyzed cross-coupling reaction, such as Suzuki or Sonogashira coupling (Figure 10A). Furthermore, the acylation of the 36 hydroxy groups on the CDs of [3]rotaxane proceeded effectively under the standard reaction conditions to yield perfectly acylated species (Figure 10B). Interestingly, the acylation of [3]rotaxane **7** at 60 °C did not proceed completely even after two weeks, although it did within 2.5 h at 80 °C. Additionally, that for a single native α-CD molecule proceeded completely at 60 °C within 2.5 h. Thus, the reaction temperature was confirmed as the key to completing the acylation reaction, probably because of the huge steric hindrance of the densely packed hydroxy groups on the [3]rotaxane species. As these modifications proceeded effectively with high yields (>90%) without any taxing purification by chromatography, a simple filtration-based purification was sufficient. This synthetic method enabled a flexible molecular design of the CD-based rotaxane framework in an easy, scalable manner.

**Figure 11:**
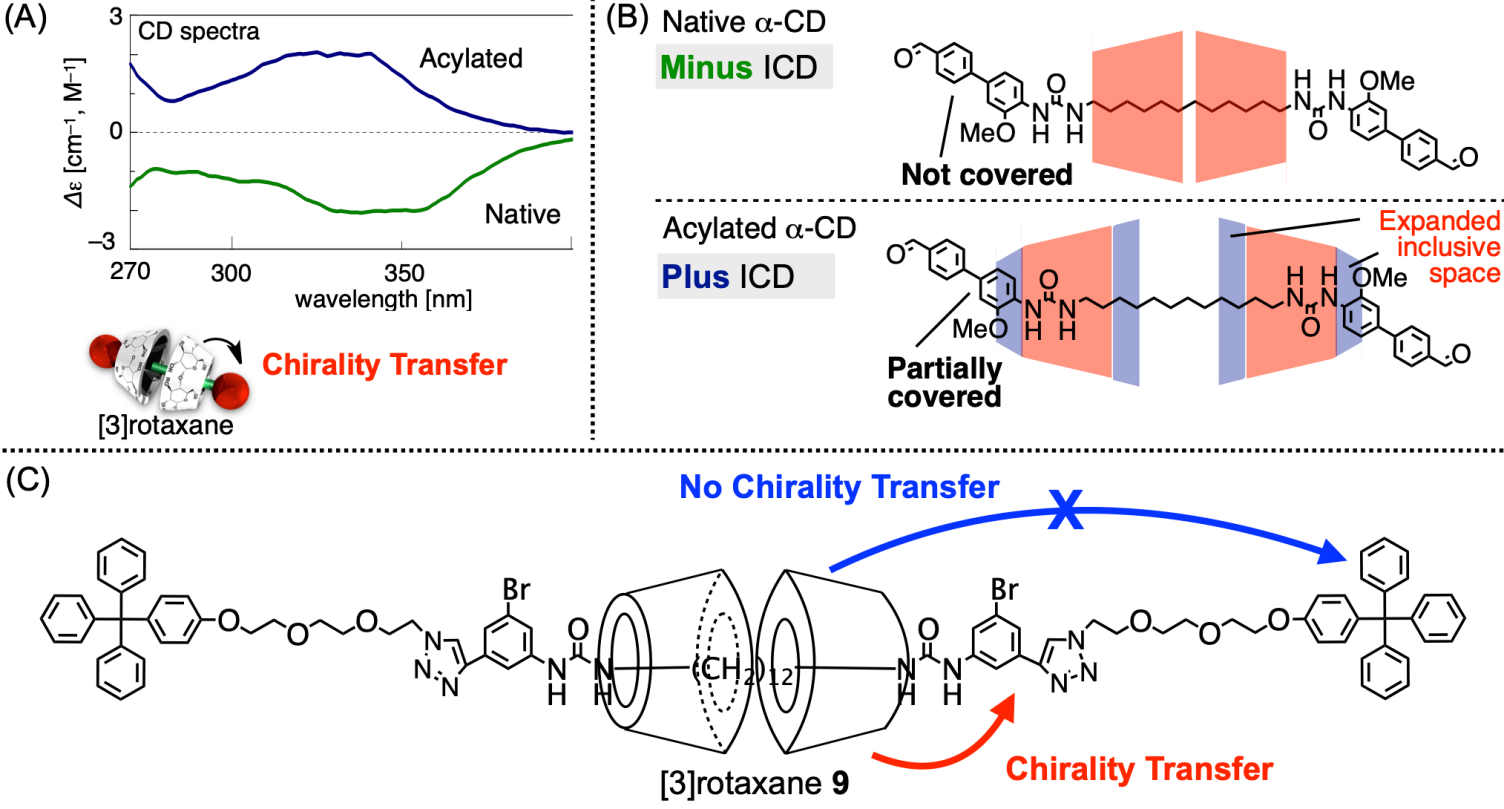
(A) ICD spectra of [3]rotaxanes bearing acylated (top) and conventional (bottom) CDs. (B) Schematic of the relationship between the plus–minus of ICD and conventional (top) or acylated (bottom) CDs. (C) Chemical structure of [3]rotaxane **9** exhibiting spacer groups.

Moreover, the obtained modified rotaxane species were evaluated by ICD analysis to further clarify their structures [74]. The results revealed that the plus and minus signs of the Cotton effect of the rotaxane changed according to the modification of the CDs: the native and methylated CD-based samples displayed the minus sign, whereas the acylated samples displayed the plus sign, and their plus-sign intensities increased in proportion to the length of the acyl chain (acetyl < propionyl < butyryl). These observations were consistent with the DFT-obtained simulated ICD spectra. Briefly, the minus Cotton effect was caused by the non-covered axle-end groups by the conventional CDs on the [3]rotaxane framework, whereas the plus effect was induced by the partially covered axle-end groups by the acylated CDs (Figure 11A,B). On the former, two CD units interact on the center of the alkyl chain via hydrogen bonding, resulting in the non-covered axle-end groups (Figure 11B, top). Meanwhile, the acylation of the hydroxy groups on the CDs expanded the cavity size along the axle, and the disappearance of the hydrogen bonding between the two CD units placed the CDs on the axle ends, causing the partial coverage of the axle ends (Figure 11B, bottom). Furthermore, [3]rotaxanes bearing spacer moieties were synthesized as the model compounds of the macromolecular [3]rotaxane. In these [3]rotaxanes, Cotton effects were only observed on the dyes near the central CD units, and the aromatic groups located far from the CDs did not display any ICD signal (Figure 11C), indicating that this ICD analysis would work as a quick indicator of the position of the wheels on the axle component, even in frameworks exhibiting extended axle-end structures.

**Figure 12:**
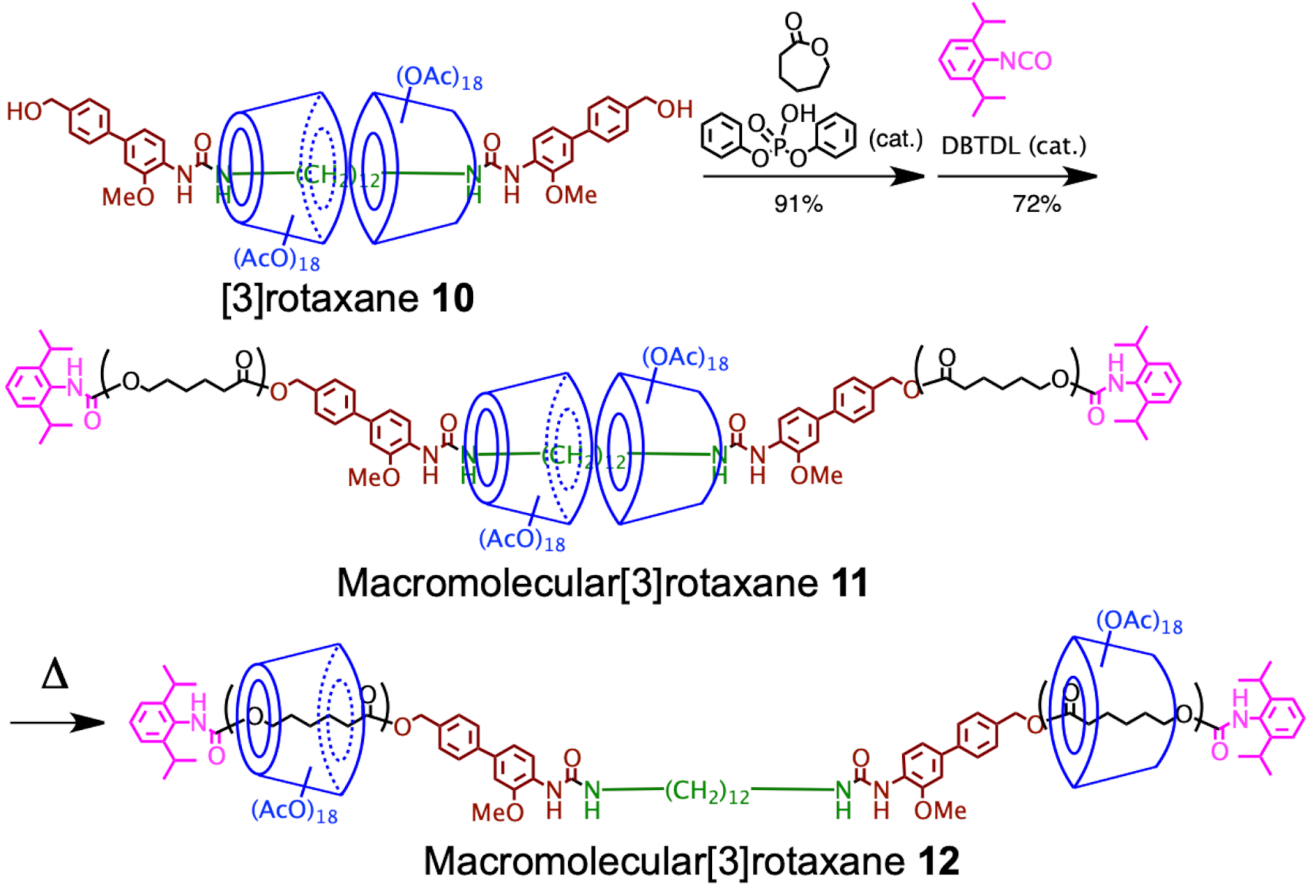
Synthesis of macromolecular[3]rotaxane via a size-complementary protocol.

Based on this molecular design, the size-complementary [3]rotaxane was integrated into the polymer main chain to synthesize macromolecular [3]rotaxane, where only two wheel components exist on the linear polymer chain [75]. Such a CD-based macromolecular rotaxane has never been synthesized owing to its synthetic complexities, supporting its innovativeness and importance. Here, [3]rotaxane diol **10** was used as the initiator of the controlled ring-opening polymerization (ROP) of ε-caprolactone in the presence of a diphenyl phosphate catalyst to introduce the polyester main chain into the rotaxane framework; the successive end-capping reactions yielded macromolecular [3]rotaxane **11** (Figure 12). In this synthesis, the hydroxy groups on CDs were completely acetylated to facilitate ROP only from the axle-end diol moieties and increase the solubility of [3]rotaxane. Further, subjecting the polymer to heat treatment induced the deslipping of CDs from the central alkyl chain on **11** to the polymer chains to freely move there (macromolecular [3]rotaxane **12**). In this deslipping, the rate-determining step was the movement of CDs over the size-complementary moiety. The movement on the polymer chain was much faster, as suggested by the same deslipping speed observed on multiple polyester lengths. Despite the deslipping behavior implemented on the polymer framework, it proceeded significantly slower than those in typical small-molecule systems probably because the multiple substituents integrated with the size-complementary moieties for the polymer-chain introduction prevented smooth deslipping. This phenomenon must be resolved in the study of CD-based macromolecular rotaxanes. However, via the structural control based on size complementarity, CD-based macromolecular rotaxane was synthesized for the first time, supporting the utility of this framework on the rotaxane-based polymer architectures with defined structures.

In summary, the structural-regulation method for CD-based rotaxanes displays a clear trend: (1) precise control but complicated framework on small molecules, and (2) easy process but challenging control on polymer systems. To overcome this trade-off, a new method has been developed using a size-complementary rotaxane framework, resulting in the first synthesis of a CD-based macromolecular [3]rotaxane. Meanwhile, since the existing size-complementary framework requires a long time for the structural transformation in the polymer system, future studies may consider the further improvement of their functions. In the next section, a more material-oriented development of the rotaxane-based polymer is described, especially from the standpoint of achieving the desired properties via the structural control of the rotaxane framework.

### Properties and material applications of rotaxane-based polymers

Polymer materials having rotaxane structures exhibit unique properties compared with conventional materials owing to the higher molecular mobility of the former [76–78]. In this regard, one of the most successful material applications is the slide ring gel, which consists of the rotaxane crosslinking structure, as a tough material or ultra-stretchable/swelling polymer [79–80]. A typical slide ring gel has a network structure of polyrotaxane, which consists of a linear polymer chain like PEG and many α-CD rings on it, meaning that the crosslinking points are made of 8 shaped two CD rings movable along the polymer main chains. Thus, the applied stress can be distributed to the entire material by the movement of the crosslinking points to avoid a stress concentration, leading to a higher toughness than the conventional materials. Since a lot of existing review articles have covered the study of slide ring gel, this review does not go into the details of this topic. Meanwhile, the structural control of this slide ring gel has been generally conducted in a rough regulation, meaning further development could be considered by the precise structural control as described in this review. As explained in the beginning, the trade-off between facile production and precise structural control is generally an important issue of rotaxane-based polymer material. In this section, multiple examples are described that studied the specific properties of the polymer material featuring the CD-based rotaxane framework by implementing a certain level of structural control at the same time.

**Figure 13:**
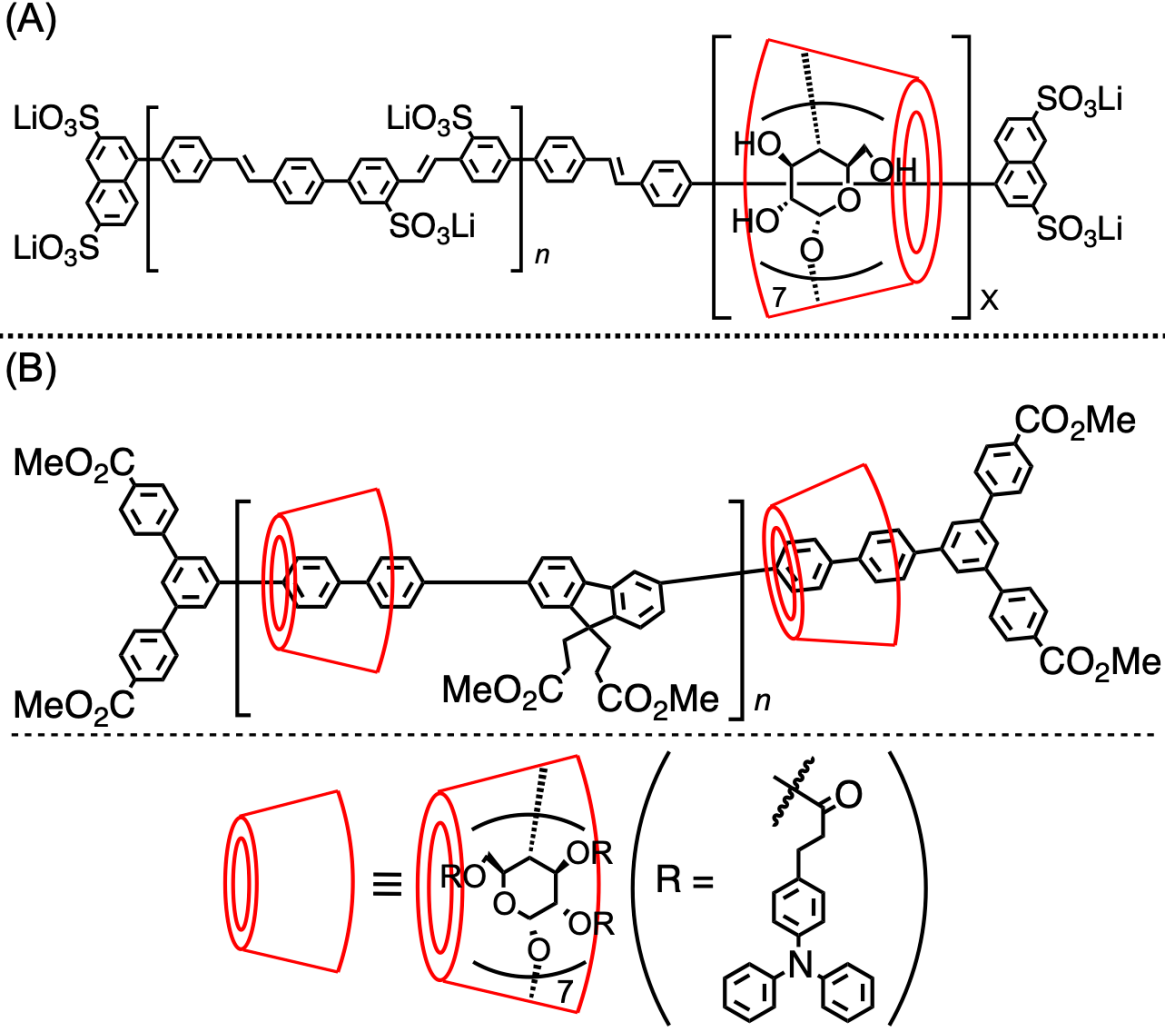
Conjugated polymer insulated by (A) β-CD. (B) Triphenylamine-substituted β-CD.

In 2013, Anderson and co-workers synthesized a conjugative polymer, which was included by β-CDs, and investigated their optical properties and thermal stabilities (Figure 13A) [81]. Polyrotaxanes were prepared with a coverage ratio of 18, 46, 64, and 71%, and the conjugative polymer without any CD coverage showed a decrease of the UV–vis absorption and its broadening at around 40–55 °C than those at 25 °C, while this spectral change became weaker according to the increase of the coverage ratio. Namely, the inclusion of CD prevented the aggregate formation of the conjugative polymer backbone, which usually induces a change in the absorption wavelength. Similarly, in fluorescence spectroscopy, the restriction of the nonradiative deactivation was caused by the inclusion structure, increasing the fluorescence intensity. This system could be described as an example in which the nature of the CD having a thickness along the cavity was effectively used on the polyrotaxane structure to improve the polymer properties. The same results would not be observed when wheel components having a small thickness are used. Moreover, this polymer can dissolve in water owing to the hydrophilicity of the CD units.

Based on the above method, a conjugative polymer containing triphenylamine- (TPA)-modified β-CD was reported (Figure 13B) [82]. In this system, the excitation energy and electron transfer were observed from the TPA to the conjugative polymer backbone included by perfectly arylated β-CD. TPA units located near the axle polymer are likely to induce an electron transfer, while those located far from the axle polymer tend to cause an excitation energy transfer. Thus, the polymer unit excited by the excitation energy transfer is located far from the TPA units, preventing quenching via electron transfer. Meanwhile, around 200 TPA units were included in one polymer molecule. The abovementioned property is induced by the nature of CD, which has many modifiable substituents (hydroxy groups) on it, besides it has a thick ring structure. The electrochemical properties of the modified CD-based conjugative polymers have been intensively studied by Terao and co-workers [83].

**Figure 14:**
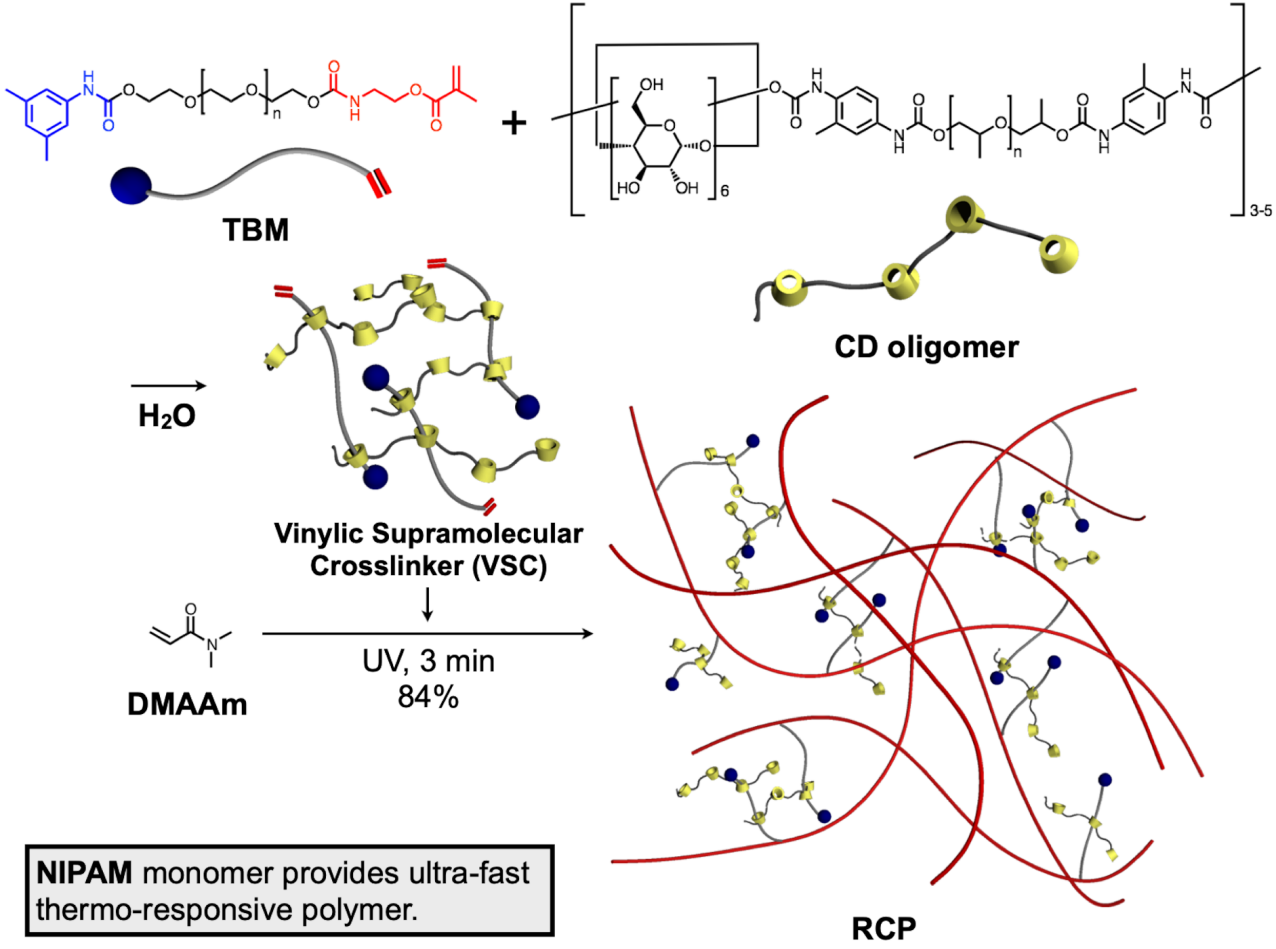
Synthesis of the VSC and successive rotaxane-crosslinked polymer (RCP) preparation.

Apart from a typical CD-based polyrotaxane network, which contains CDs and linear polymer chain-based polyrotaxane as a major content of the material, Takata and co-workers developed the rotaxane crosslinker, which can be integrated as a small content on vinyl polymer synthesis to afford vinyl polymers having a certain domain of polyrotaxane crosslinking points (Figure 14) [84]. In this system, the crosslinker was named the vinylic supramolecular crosslinker (VSC), which was made of an axle component having a polymerizable vinylic group and a bulky end-capping group on the other side, and α-CD oligomer having around 3–5 α-CDs linked by a PPG linker with urethane bonds as the wheel component. This VSC was easily synthesized and exhibited a rotaxane crosslinking nature; hence, the resulting vinyl polymer, e.g., polyDMAAm crosslinked by VSC, showed enhanced stretchability induced by rotaxane crosslinking. Moreover, when it was applied to a thermo-responsive polymer like poly(*N*-isopropyl acrylamide) (NIPAM), the thermo-responsive nature became much faster than the system without VSC. This fast responsiveness was induced by the dynamic rotaxane crosslinking structure. This method can be applied in various vinyl polymer systems, and induces the rotaxane crosslinking nature to the resulting network polymer, suggesting its robustness and utility. However, the VSC structure contains many ambiguities, such as the coverage ratio of one polymer chain, the directions of the wheels, and the number of wheel units on one CD oligomer. To control the properties of the network polymer, a more precise design of the rotaxane crosslinker was desired.

**Figure 15:**
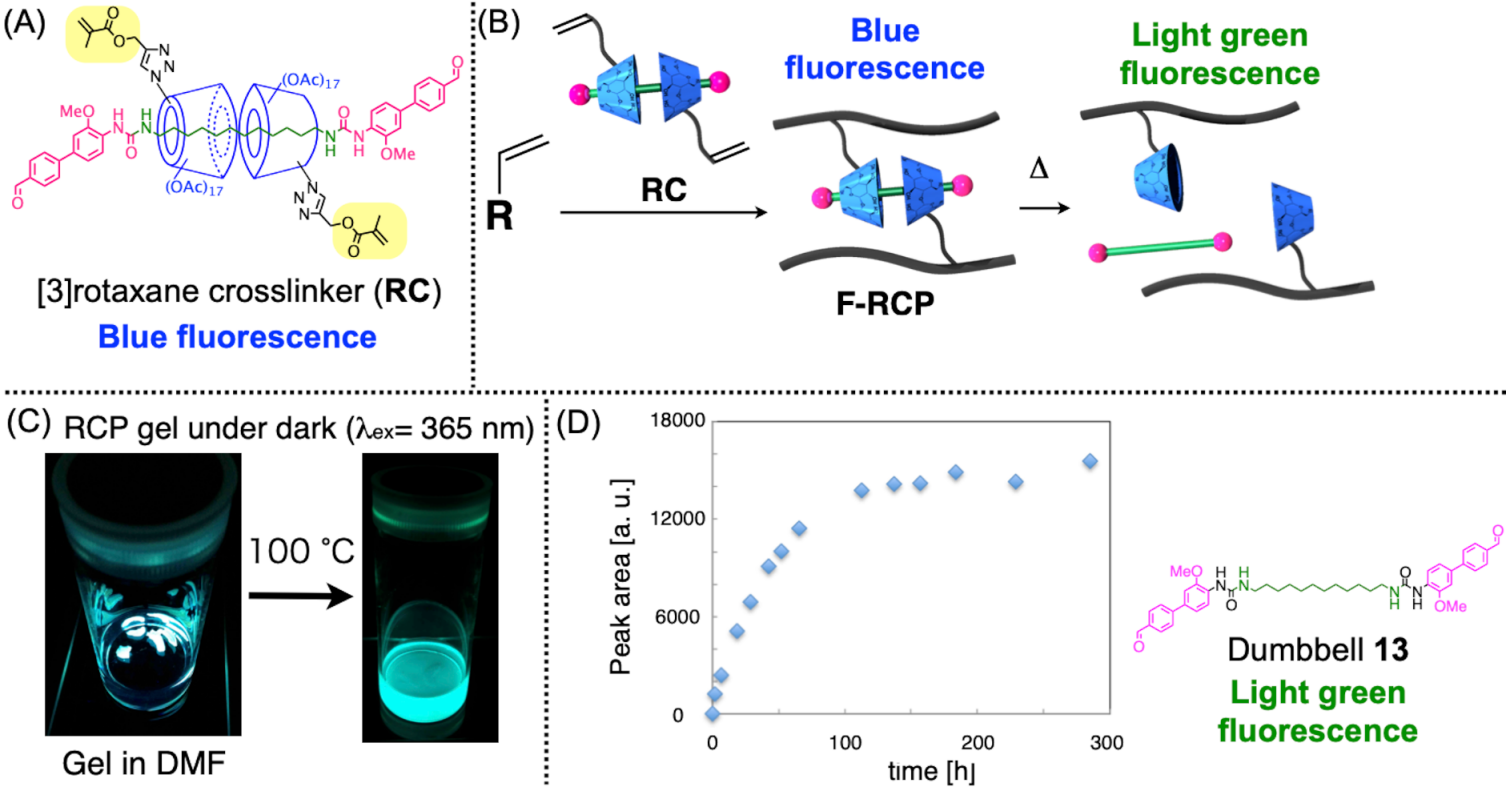
(A) Chemical structure of the [3]rotaxane crosslinker (RC). (B) Schematic of the synthesis and de-crosslinking of the rotaxane-crosslinked polymer (RCP). (C) Photo of RCP in DMF in the dark before (left) and after (right) heating. (D) Time-course of the fluorescence intensity of the dumbbell **13** released according to the de-crosslinking of RCP. Figure 15C was adapted from [85], Y. Akae et al., “Cyclodextrin-Based [3]Rotaxane-Crosslinked Fluorescent Polymer: Synthesis and De-Crosslinking Using Size Complementarity”, *Angew. Chem., Int. Ed.,* with permission from John Wiley and Sons. Copyright © 2018 Wiley-VCH Verlag GmbH & Co. KGaA, Weinheim.

To prepare a structure-defined CD-based rotaxane crosslinker, we synthesized a CD-based [3]rotaxane via the urea end-capping method with mono-substituted α-CD (Figure 15A) [85]. Since the urea end-capping method affords [3]rotaxane having a defined structure, i.e., defined number (two) and directions (head-to-head) of wheel components, in high yields, it is suitable for use in crosslinker synthesis. The CD unit has defined one methacryl moiety on its sixth position, providing two polymerizable vinyl groups on [3]rotaxane. Moreover, since the remaining 34 hydroxy groups on the two CD units are easily modified by acylation, the hydrophilicity of the rotaxane crosslinker (RC) could be tuned in this manner. This [3]rotaxane crosslinker RC was added 0.5 mol % on the standard free radical polymerization of vinyl monomers to afford rotaxane-crosslinked polymer (RCP) (Figure 15B). Similar to the VSC-based system, this crosslinker can be applied to various vinyl polymerizations, and the resulting RCP showed properties depending on the monomers used. Meanwhile, since the size-complementary framework was integrated with this crosslinker, heating induced the decomposition of the rotaxane crosslinking moiety, resulting in the de-crosslinking of the entire network structure without damaging the trunk polymer backbone. Furthermore, when the fluorescence active moiety was integrated with the dumbbell structure, this de-crosslinking process was monitored by the time-course change of the fluorescence (Figure 15C,D). Namely, the fluorescence of the initial RCP in DMF was observed as a blue emission by the naked eye, while according to the degradation, the dumbbell species spread throughout the solution and the [3]rotaxane structure disappeared, resulting in the light green color fluorescence of the entire solution derived from the release of the dumbbell **13** after the decrosslinking. This fluorescence wavelength change was induced by the solvation of the dye moieties. Since the dumbbell contains the push-pull type substituents on the biphenyl structure, this dye showed solvatochromism, meaning the introduction of CD units affects the fluorescence in a similar manner to the solvent, probably because two CD units have densely placed many (36) substituents on them. Thus, PAc-α-CD-based [3]rotaxane showed blue-shifted fluorescence compared with the dumbbell **13**, which was observed before and after the de-crosslinking of the gel. In addition to the qualitative observation by the naked eye, the quantitative kinetic analysis of this decrosslinking was conducted by fluorescence spectroscopy. Such a quantitative study became possible thanks to the defined rotaxane structure, which supported the importance of the defined structure construction on rotaxane-based polymer.

**Figure 16:**
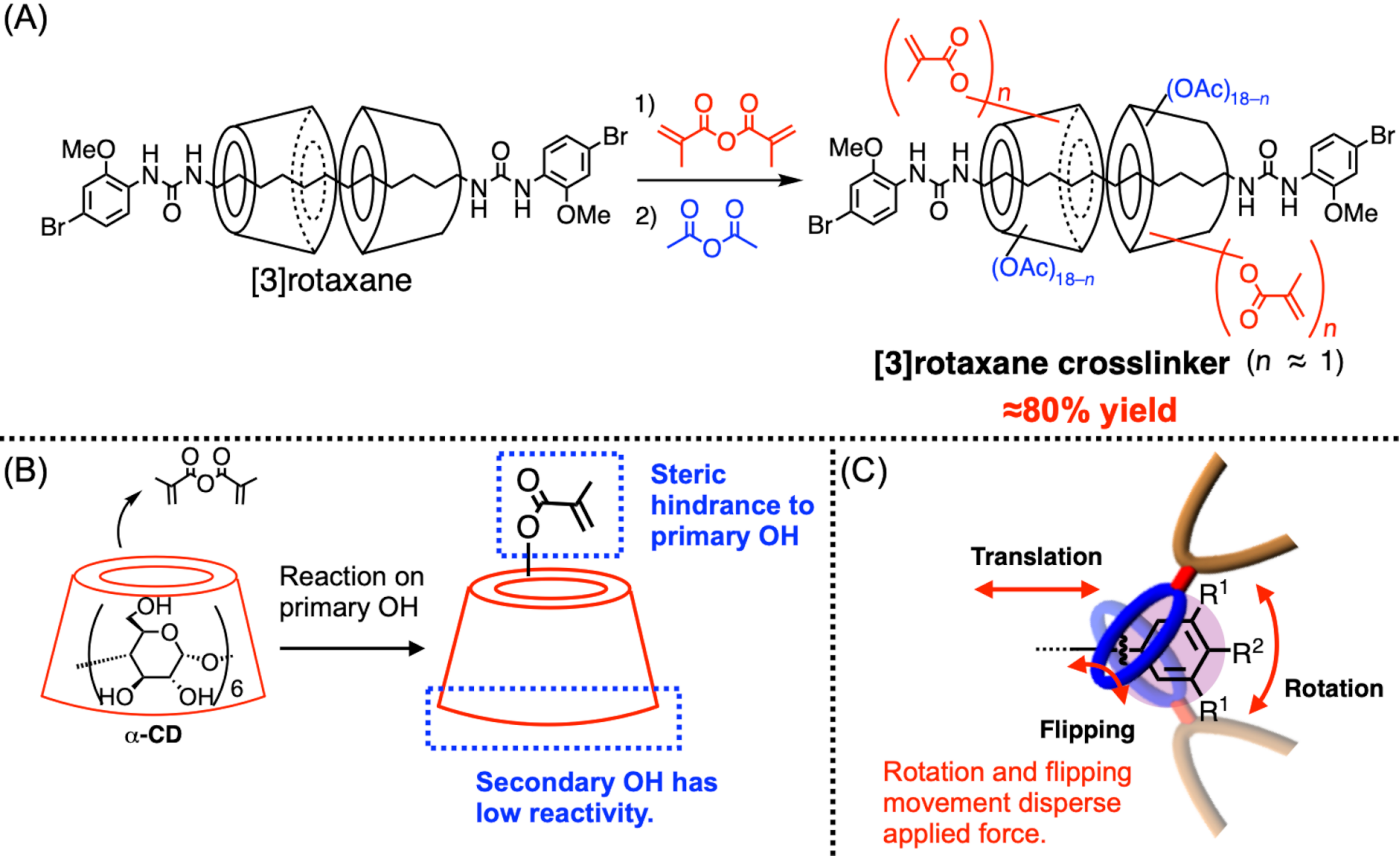
(A) Random vinylation of the CD-based [3]rotaxane; (B) Schematic of the reaction between α-CD and methacrylic anhydride. (C) three movement modes on the rotaxane crosslinking point (translation, rotation, and flipping).

Although the [3]rotaxane crosslinker having a completely defined structure is beneficial for precise structure–property control, mono-substituted CDs are well known for their low-yield production [31,85]. This is because at least six glucose units exist in the usual CD molecules, causing the necessity of the purification step to isolate only the mono-substituted product from the mixture containing others, such as two- or three-substituted species or the starting material (CD) itself. For example, a typical mono-substituted α-CD synthesis is conducted by tosylation and a successive functional group transformation, and this tosylation often results in around 10% yield owing to the above reason. Because the mono-substituted α-CD is also useful in other stimuli-responsive material production [86], it’s easier synthesis was highly desired. Therefore, we developed a random modification of α-CD via acylation with methacrylic anhydride, which provided roughly one substituent introduction in α-CD (Figure 16A) [87]. In this reaction, owing to the steric hindrance caused by the first introduction of the methacryl group on the primary alcohol at the sixth position (the narrower rim side), the further reaction on the narrower rim side of the CD was disturbed, while the secondary alcohol groups on the wider rim side of the CD could not react owing to their low reactivities (Figure 16B). Thus, the introduction of the methacryl group was roughly controlled as one substituent even in a random manner in high yields (84–97%). Here, the reaction temperature was the key for control, since a higher temperature above 80 °C induced the introduction of multiple substituents, meaning the above factors did not work properly at the high reaction temperature. Meanwhile, at 60 °C, only 140 mol % of the methacryl group was introduced to α-CD, even when 6700 mol % of the reagent was used for the reaction (1800 mol % is the theoretical maximum), suggesting the impact of the temperature in this system. By applying this random modification to the [3]rotaxane **7**, the [3]rotaxane crosslinker was also synthesized with approximately 1.3 methacryl units per CD in a 78% yield, which successfully afforded the corresponding RCPs. When it comes to industrial material production, a certain deviation from the desired modification number is generally acceptable to reduce the production costs, suggesting the industrial potential of this random modification protocol. As suggested by the recent study on the RCP made of crown ether-based [3]rotaxane crosslinker, the force distribution effect worked sufficiently to toughen RCPs, even when the translation movement of the wheel components along the axle component could not work well, because the rotation or flipping movement dispersed the applied force effectively (Figure 16C) [88]. Although most of the rotaxane-based polymer network studies have focused on the translation movement as a central role in the toughening effect of the rotaxane crosslinking, the contribution of the rotation or flipping movement will be further studied in the future. In this regard, the CD-based structure-defined [3]rotaxane and the facile random modification of CD would contribute to the relevant study.

**Figure 17:**
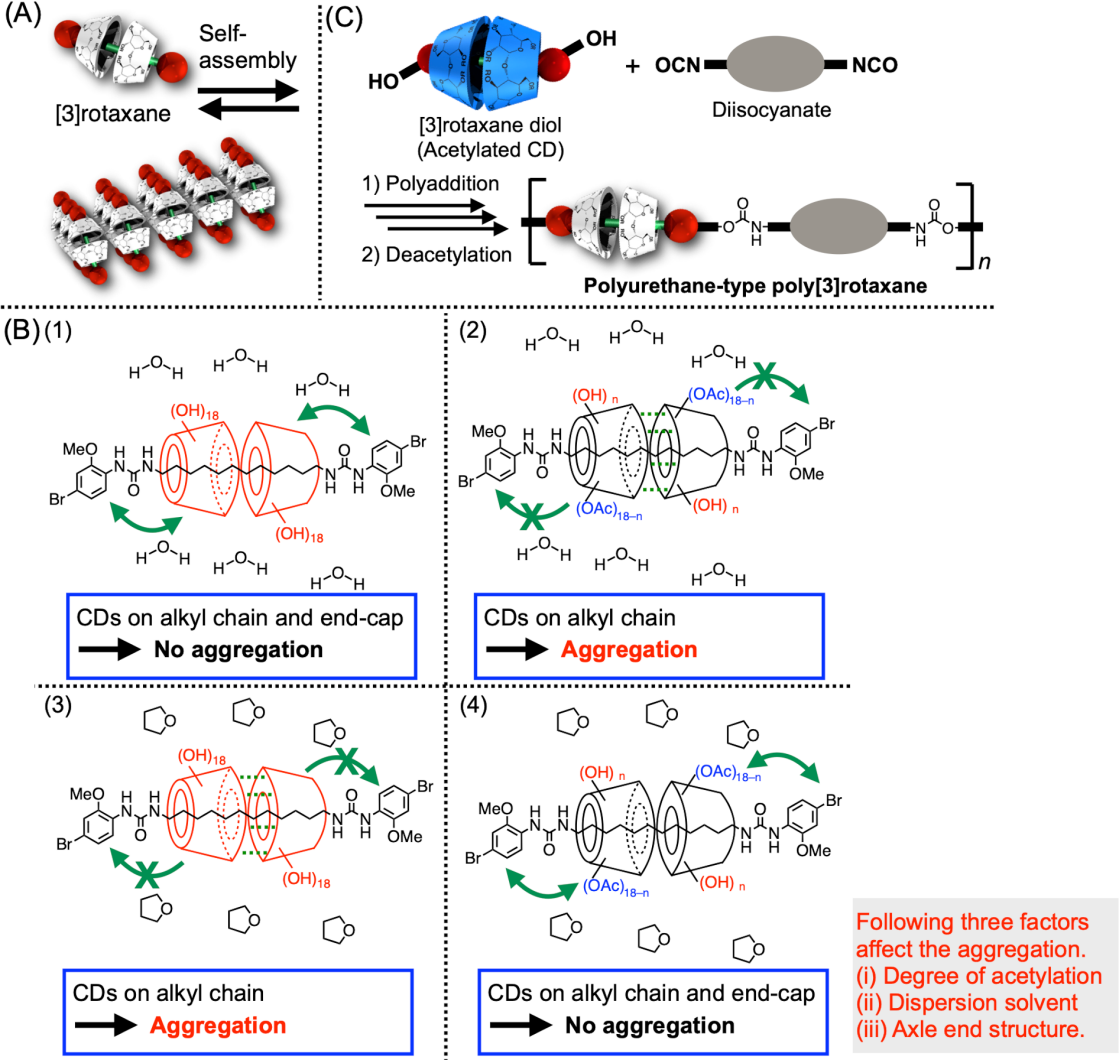
(A) Aggregation of CD-based [3]rotaxane. (B) Schematic of the plausible mechanism of the aggregation behaviors of (1) native CD-based [3]rotaxane in H_2_O, (2) partly acetylated CD-based [3]rotaxane in H_2_O, (3) native CD-based [3]rotaxane in THF, and (4) partly acetylated CD-based [3]rotaxane in THF. (C) Synthesis of polyurethane-type poly[3]rotaxane.

Besides the highly dynamic nature of the rotaxane framework, CD-based polyrotaxane is known to form an aggregation structure by the formation of hydrogen bonds among the CD units [89–93]. Owing to the inclusion structure on the polyrotaxane, it is likely to form a hexagonal packing-type crystalline structure, which is different from that of a single CD molecule. Such an aggregation behavior of polyrotaxane induces the increase of the mechanical strength of the material or the construction of a nanosheet, which is beneficial for material application. Meanwhile, CD-based polyrotaxane requires various synthetic demands, as discussed in the previous sections, resulting in the limited applicability of this aggregate formation. To solve this issue, we used a CD-based [3]rotaxane unit as an aggregation unit instead of a CD-based polyrotaxane. As described above, the [3]rotaxane unit can be flexibly designed and integrated with the polymer framework, which would overcome the synthetic limitation of the conventional polyrotaxane design, e.g., the most popular system consisted of PEG and many α-CD units. Thus, the aggregation behavior of the [3]rotaxane molecule without the polymer framework was first studied (Figure 17A) [49]. As a result, it was clarified that [3]rotaxane species also formed a hexagonally aggregated structure similar to the polyrotaxane system, even though only two CD units were located on one molecule. The aggregation behavior was highly affected by the solvent type, axle-end structures, and the degree of acetylation of the CD units. This is understandable considering that the main driving force of the inclusion formation of CD is a hydrophobic interaction, which is highly affected by the above factors. The details were checked by the original article, but in short, the interaction among the hydrophilic native CD unit, hydrophobic partly acetylated CD, and axle-end groups in the given solvent dominates if the aggregation is formed or not (Figure 17B). As reported recently, this [3]rotaxane unit was integrated with the polyurethane-type poly[3]rotaxane synthesis by the polyaddition reaction of [3]rotaxane diol and various di-isocyanate comonomers (Figure 17C) [94]. In this synthesis, [3]rotaxane was first acetylated to protect the hydroxy groups on the CDs and deprotected after the polyaddition. Thus, the polyurethane framework could be flexibly designed as the main chain of CD-based poly[3]rotaxane, which has been difficult by conventional synthesis. The morphology of poly[3]rotaxane changed significantly between before and after the deacetylation of the CD units, but the aggregation was not as effectively induced as in the case of the small [3]rotaxane molecule, probably because the integrated polymer framework prevented the flexible placement of [3]rotaxane units to form the aggregation, suggesting that a further suitable polymer backbone structure should be studied in the future. For example, various polymer main chain frameworks can be attached to [3]rotaxane diol using a recently developed synthetic method, which easily provides polymers featuring isocyanate groups on one side of their ends [95–97]. Moreover, polymerization of comonomers having pseudo[2]rotaxane structure or further modification after the polyrotaxane formation have been also reported to address diverse polymer main chain types [98–101].

As described above, besides the very popular toughening effect by rotaxane crosslinking, the structural control of the rotaxane-based polymer results in unique property induction on, e.g., energy/electron transfer, hydrophilicity/hydrophobicity, thermo-responsiveness, fluorescence, and aggregation. The defined rotaxane structure enables detailed quantitative observation as demonstrated by the time-course change of the fluorescence of RCP, suggesting its importance for high-resolution property analysis of the polymer material. Meanwhile, a property induced in the small rotaxane molecule is not always equally integrated with the polymer system as reported on the aggregation behavior. It would mainly depend on the nature of the intended property, whether the property transfer from the small molecule to the polymer system works or not. When the intended property is based on the intramolecular interaction, the rotaxane unit in the polymer framework would work as same as the small rotaxane molecule, while in the case of the intermolecular interaction, the polymer structure affects a lot on this issue. Considering the unique properties reported in the rotaxane-based polymer featuring CD wheels, further development for its precise structural control to study remaining unclear issues could be a promising approach to reach out unaddressed properties by conventional materials.

## Conclusion

As mentioned at the beginning of this paper, implementing precise structural control on CD-based rotaxane systems is not as easy as implementing it on rotaxane systems exhibiting other wheel components. Meanwhile, this review reveals that the unique properties of the CD molecule exert a synergetic effect with the combination of the rotaxane framework and polymer material to introduce various exotic characteristics specifically induced in rotaxane-based materials featuring CD wheels, for instance, the changing/tuning of their hydrophilic/hydrophobic natures, the introduction of chirality, the formation of aggregates, and the introduction of electrochemical insulation via the thickness of CD. Thus, in addition to the inexpensiveness of the CD molecule, a great potential for new science can be derived from CD-based rotaxanes, which could be addressed by the further precise control of their structures. To implement a higher resolution of the structural control, the relevant studies must be approached from the aspects of rotaxane-based polymer and small rotaxane molecules, as they interact. Thus, we have been developing an original system of a CD-based [3]rotaxane framework as an option for achieving a correlation between the inexpensive production and defined structures for rotaxane-based polymer materials. Although this is a still challenging task, it is essential to direct the study of CD-based rotaxanes to a precise synthesis rather than to stay in the currently dominant rough structural control to meet the existing demand for tailored-material production.

## Data Availability

Data sharing is not applicable as no new data was generated or analyzed in this study.
